# Systematic pharmacology-based strategy to investigate the mechanism of beta-sitosterol for the treatment of rheumarthritis

**DOI:** 10.3389/fgene.2024.1507606

**Published:** 2024-12-04

**Authors:** Xiaodong Wang, Jingxin Mao

**Affiliations:** ^1^ Department of Medical Technology, Chongqing Medical and Pharmaceutical College, Chongqing, China; ^2^ College of Pharmaceutical Sciences, Southwest University, Chongqing, China

**Keywords:** rheumarthritis, beta-sitosterol, experimental verification, systematic pharmacology, mechanism

## Abstract

**Objective**: *β-*Sitosterol, which is derived from *Vladimiriae Radix* (VR), is used for the treatment of rheumatoid arthritis (RA), but the pharmacological mechanisms through which *β-*sitosterol affects RA have not been fully elucidated.

**Methods**: Through the Traditional Chinese Medicine Systems Pharmacology and Analysis (TCMSP), PubChem, SwissTargetPrediction, GeneCards, DisGeNET, and OMIM databases, “*β-*sitosterol-RA”-related genes were obtained, and a target protein interaction network (protein–protein interaction [PPI]) was constructed. Gene Ontology (GO) and Kyoto Encyclopedia of Genes and Genomes (KEGG) pathway enrichment analyses were carried out for the intersecting genes. Discovery Studio 2019 software was used to perform molecular docking on *MMP9*, *CASP3*, *HSP90AA1*, *SRC*, *EGFR*, and *ALB* genes. *β-*Sitosterol was co-cultured with MH7A cells in three experimental groups: control group (DMSO), positive drug group (methotrexate, 80 μmol/L), and drug intervention group (10, 20, 40, 80, and 160 μmol/L *β-*sitosterol). The CCK8 method was used to investigate the inhibitory effect of *β-*sitosterol on the proliferation of MH7A cells. RT-PCR was used to analyze the mRNA expression of the abovementioned core targets.

**Results**: A total of 41 genes associated with *β-*sitosterol and RA were obtained, mainly involving the FoxO signaling pathway and PI3K/AKT signaling pathway. The molecular docking results suggested that *β-*sitosterol could bind effectively to six core targets. The experimental results showed that *β-*sitosterol could significantly inhibit the excessive proliferation of MH7A cells (*p<* 0.05). The RT-PCR results showed that the expression of *MMP9*, *HSP90AA1*, *SRC*, *EGFR*, and *ALB* core genes in the control group was significantly upregulated, while the *CASP3* gene was downregulated. Compared to the control group, the mRNA expression of *MMP9*, *HSP90AA1*, *SRC*, *EGFR*, and *ALB* decreased (*p<* 0.01), while the apoptosis-related gene *CASP3* increased in both the drug intervention (80 μmol/L *β-*sitosterol) and positive drug groups (80 μmol/L methotrexate).

**Conclusion**: Hence, *β*-sitosterol could contribute to the inhibition of RA by modulating cell proliferation and regulating the aforementioned six core proteins, potentially through the regulation of the FoxO and PI3K/AKT signaling pathways.

## 1 Introduction

Rheumatoid arthritis (RA) is a chronic and systemic autoimmune disease characterized by the symmetrical synovial inflammation of joints and the destruction of bone and cartilage structures (Rashed, 2020). Its clinical manifestations include erosive symmetric polyarthritis, joint pain, numbness, and gradual destruction of articular cartilage and bone, eventually leading to joint deformity and loss of function (Van Tuyl et al., 2014; Chemin et al., 2016). The disease is widely distributed and extremely difficult to cure, with a high rate of injury and disability (Verstappen et al., 2004). The pathogenesis of RA is complex. At the same time, angiogenesis, synovial hyperplasia, bone erosion, and cartilage tissue damage caused by the inflammatory environment result in the destruction of the entire joint (Moran-Moguel et al., 2018). It was reported that RA may occur in all age groups; the incidence rate is between 0.5% and 1%, and it is more common in women (Suzuki and Yamamoto, 2015). Currently, the drugs used to treat RA are commonly divided into non-steroidal anti-inflammatory drugs, anti-rheumatic drugs to improve the condition, glucocorticoids, immunosuppressants, biological agents, and Chinese herbal medicine. The first five categories of drugs have single efficacy and many adverse reactions, which greatly limit their clinical application (Rein and Mueller, 2017). Traditional Chinese medicine (TCM) is rich in resources and has accumulated rich experience in folk medicine. Its overall action characteristics of multi-components, multi-pathways, and multi-targets have significant advantages in the treatment of RA. The search for active anti-RA drugs from TCM has become the focus of many researchers engaged in basic and clinical research.

*Vladimiriae Radix* (VR), recorded in the Pharmacopoeia of the People’s Republic of China (2020), is the dried root of the Compositae plant *Vladimiria souliei* (Franch.) Ling or *V. souliei* (Franch.) Ling var. cinerea Ling (National Pharmacopoeia Committee, 2020). The root of VR is used as traditional medicine for its efficacy in promoting “qi,” relieving pain, and warming the stomach (National Pharmacopoeia Committee, 2020). In clinical practice, VR is mainly used to treat intestinal and digestive diseases (Mao et al., 2022). In recent years, with extensive research, a variety of pharmacological effects of VR have been gradually discovered, among which the anti-inflammatory effect has been repeatedly reported (Mao et al., 2018). The extract of VR may effectively alleviate the symptoms of inflammation in mice caused by xylene and acetic acid and has anti-inflammatory and analgesic effects (Mandal et al., 2020). In addition, our previous studies also revealed that VR and its extract exhibit anti-cancer activity (Mao et al., 2019). Beta *(β)*-sitosterol is a major sterol of VR. It is a plant sterol with a four-ring and three-label compound, which is widely found in many plants and Chinese herbal medicine. Its structure is similar to that of cholesterol (Rashed, 2020). *β-*Sitosterol is mostly white powder or needle-shaped crystals at room temperature, with a high melting point (130°C–140°C). It is insoluble in water and soluble in some organic solvents, such as ethanol, ethyl acetate, and dimethyl sulfoxide. The structure of *β-*sitosterol is similar to that of cholesterol, which has been reported for many biological activities (Gupta, 2020). It can inhibit the absorption of cholesterol by the human body, thereby reducing the cholesterol content in the human body. At the same time, it may inhibit the biosynthesis of cholesterol, preventing the occurrence of high-cholesterol diseases in many ways (Shi et al., 2011). Modern pharmacological research confirmed that *β-*sitosterol also exhibits anti-oxidant (Vivancos and Moreno, 2005), anti-cancer (Novotny et al., 2017), anti-inflammatory (Liao et al., 2018), and immune activity (Bouic, 2001) effects. Recent research findings suggest that *β-*sitosterol may play an important role in the treatment of RA (Qian et al., 2022), which has attracted more attention.

Network pharmacology is a method based on system biology and multi-directional pharmacology theory to predict the effective components and molecular mechanisms of drugs from a holistic perspective by building a “drug–target–disease” interaction network (Jiang et al., 2022). The drug action network is mapped to the human disease gene network to explore the interaction between drugs and diseases. Therefore, the present study applied network pharmacology, molecular docking technology, and experimental verification to further understand the action targets and treatable diseases, which would provide an effective cure for RA using *β-*sitosterol.

## 2 Network pharmacology analysis and experimental verification

### 2.1 Network pharmacology database

Traditional Chinese medicine Systems Pharmacology and Analysis (TCMSP) (http://tcmspw.com/tcmsp.php); PubChem (https://pubmed.ncbi.nlm.nih.gov/); SwissTargetPrediction (http://www.swisstargetprediction.ch/); GeneCards (http://www.gencards.org/); DisGeNET (http://www.disgenet.org/); OMIM (http://omin.org); VennDiagram (http://bioinformatics.psb.ugent.be/webtools/Venn/); UniProt (https://www.uniprot.org/); STRING 11.0 (https://string-db.org/); and PDB databases (https://www.rcsb.org/) were used in this study (Table 1).

**TABLE 1:** Database/website used in the experiment.

Database/website	Website address
Traditional Chinese Medicine Systems Pharmacology and Analysis (TCMSP) database	http://tcmspw.com/tcmsp.php
PubChem database	https://pubmed.ncbi.nlm.nih.gov
SwissTargetPrediction website	http://www.swisstargetprediction.ch
GeneCards database	http://www.gencards.org
DisGeNET database	http://www.disgenet.org
OMIM database	http://omin.org
VennDiagram website	http://bioinformatics.psb.ugent.be/webtools/Venn
UniProt database	https://www.uniprot.org
String 11.0 database	https://string-db.org
PDB database	https://www.rcsb.org

### 2.2 Extraction of target genes

Using “*β-*sitosterol” or “beta-sitosterol” as the keyword, the TCMSP, PubChem, and SwissTargetPrediction databases were searched to obtain the protein target of *β-*sitosterol. UniProt was utilized to convert the proteins corresponding to the above targets into species human genes and construct a database of *β-*sitosterol and its core target genes.

### 2.3 Screening of *β*-sitosterol anti-RA-related targets

RA-related targets were searched through GeneCards, DisGeNET, and OMIM databases. The keyword “rheumatoid arthritis” or “RA” was used to search for the targets related to RA reported in the current research, and then the targets obtained from the database were reorganized, with the duplicates deleted. The target of *β-*sitosterol and the target related to RA were input, and the intersection of the active ingredient target and disease target was sorted and defined as the potential target of *β-*sitosterol against RA. GeneCards, DisGeNET, and OMIM databases were searched for RA-related targets, and TCMSP, PubChem, and SwissTargetPrediction databases were searched for compounds.

### 2.4 Component screening of common target genes of diseases

The two groups of targets screened were input based on the relevant database of “*β-*sitosterol–RA” into the online mapping tool VennDiagram to draw the Venn map, and the common target genes of “component–disease” were obtained.

### 2.5 “Component–target” network construction and analysis

The interaction network of *β-*sitosterol and its related targets was constructed using Cytoscape 3.7.2 software.

### 2.6 Construction of the protein–protein interaction network

The protein–protein interaction (PPI) network was analyzed using the STRING 11 database, the protein interaction relationship was obtained, and Cytoscape 3.7.2 software was used to draw the target PPI network. Following a previously established study method (Xu et al., 2022; Wang et al., 2022; Huang et al., 2023), the “Network Analyzer” module was used to analyze the network and determine the relationship between potential targets of RA in *β-*sitosterol treatment. The median values (cut-off values) of three parameters, namely, degree value (>23), betweenness centrality (>0.00132981), and closeness centrality (>0.51145038), were taken as reference indicators. Among them, the degree value was considered the most important indicator. The six highest-degree targets were selected and imported into Cytoscape 3.7.2 software, and the PPI of core targets was built.

### 2.7 Gene Ontology function and enrichment analysis of the Kyoto Encyclopedia of Genes and Genomes pathway

R 4.0.4 software was used to analyze the Gene Ontology (GO) function and Kyoto Encyclopedia of Genes and Genomes (KEGG) pathway enrichment of potential anti-RA targets of *β-*sitosterol. GO function enrichment analysis mainly includes the biological process (BP), cell component (CC), and molecular function (MF). The first 20 results of the GO function enrichment analysis were screened and visualized using a bubble diagram. The first 20 channels of the KEGG pathway enrichment analysis were screened and visualized using histograms. To validate the anti-RA mechanism of *β*-sitosterol across the key targets, the KEGG mapper functional analysis was utilized to mark the target genes on the pathway associated with RA.

### 2.8 Method of molecular docking

As a small-molecule ligand, *β-*sitosterol was derived from Chem3D 19.0 software, and its lowest energy calculation was saved in “mol2” format. The core target protein, acting as the receptor, was downloaded from the PDB database in the “PDB” format with a three-dimensional structure (the core target protein is a human protein, with a resolution and preference for structures containing original ligands). The data were imported into Discovery Studio 2019 software to conduct molecular docking between the core target protein and *β-*sitosterol (hydrogenation, dehydration, etc.) and output 2D and 3D structure diagrams.

### 2.9 Experimental verification

#### 2.9.1 Extraction and isolation

Air-dried VR rhizome powder (11.0 kg) was extracted by overnight soaking with 95% ethanol at room temperature. The ethanol extract was evaporated under vacuum conditions to obtain a semi-solid (a measure of 1.12 kg), which was then suspended in water and partitioned sequentially with petroleum ether, ethyl acetate, and n-butanol successively. The ethyl acetate solution was concentrated to obtain 296 g of residue, which was further subjected to silica gel column chromatography (100–200 mesh, 70 cm). Separation was performed using a 10-cm inner-diameter mixture of petroleum ether and ethyl acetate (99:1–10:1), resulting in a total of 16 fractions (A to P). *β-*Sitosterol was derived from fraction F (7:3) with a purity of over 85% and identified *via* spectroscopic methods (^1^H-NMR, ^13^C-NMR, and UCMS) (Supplementary Figures 1–3).

#### 2.9.2 Reagents and equipment

Dulbecco’s modified Eagle’s medium (DMEM), high-sugar medium, fetal bovine serum (FBS), penicillin/streptomycin solution, dimethyl sulfoxide (DMSO), 3-(4,5)-dimethylthiazol (-z-y1)-3,5-di-phenytetrazolium bromide (MTT) assay kit, TRIzol reagent, SP6 High-Yield RNA Transcription Kit (Beyotime Biotechnology, China), BeyoFast™ SYBR Green qPCR Mix (Beyotime Biotechnology, China), methotrexate (Shanghai Shangyao Xinyi Pharmaceutical Co., Ltd., China), bicinchoninic acid (BCA) protein concentration determination kit, radio immunoprecipitation (RIPA) lysis buffer assay kit (Tiangen Biotech (Beijing) Co., Ltd., China), ECL chemiluminescence substrate (Shanghai Tianneng Life Science Co., Ltd.), rabbit anti-matrix metallopeptidase (MMP) 9 monoclonal antibody, rabbit anti-caspase (CASP)-3 monoclonal antibody, rabbit anti-heat shock protein 90 alpha family class A member (HSP90AA1), monoclonal antibody, rabbit anti-steroid receptor coactivator (SRC) monoclonal antibody, rabbit anti-epidermal growth factor receptor (EGFR) monoclonal antibody, rabbit anti-albumin (ALB) monoclonal antibody (Wuhan Proteintech Group, Inc), rabbit anti-glyceraldehyde-3-phosphate dehydrogenase (GAPDH) monoclonal antibody (batch number: 10021787; Wuhan Sanying Biotechnology Co., Ltd.), and HRP-labeled goat anti-rabbit IgG (H+L) (Wuhan Proteintech group, Inc) were used. *β-*Sitosterol was derived from VR. A 3111 CO_2_ cell incubator (Thermo Fisher, United States), 7500 Fast Real-Time PCR System (Thermo Fisher Scientific, United States), TD5A-WS low-speed desktop centrifuge (Changsha Xiangyi Centrifuge Instrument Co., Ltd, China), CJ-20 purification workbench (Tianjin Taist Instrument Co., Ltd, China), XDS-1B inverted microscope (Chongqing Optoelectronic Instrument Co., Ltd, China), MK3 automatic microplate reader (Thermo Fisher, United States), and Bio-Rad C1000 Polymerase Chain Reaction (PCR) Detector (Bio-Rad, United States) were used.

#### 2.9.3 Cell culture

The human rheumatoid arthritis fibroblast synovial cell line (MH7A) was purchased from Generol Biological Co., Ltd. MH7A cells were cultured in DMEM high-sugar medium containing 20% fetal bovine serum at 37°C in a 5% CO_2_ incubator with a humidity of 100%. When the cells grew to more than 90% of the flask, they were routinely subcultured, and 10 passages of cells were taken for the experiment.

#### 2.9.4 MTT assay kit

The MTT method was used to detect the effect of *β-*sitosterol on the proliferation and toxicity of MH7A cells. The test was divided into the control group (DMSO), positive drug group (methotrexate, 80 μmol/L), and drug intervention group (*β-*sitosterol, 10, 20, 40, 80, and 160 μmol/L, respectively). The doses were chosen for further research based on previous studies (Cheng et al., 2015; Zhou et al., 2016; Zuo and Ma, 2024; Zhu et al., 2023). Among the groups, 10 μL DMSO was added to the control group, 10 μL methotrexate at a concentration of 80 μmol/L was added to the positive drug group, and 10 μL *β-*sitosterol at concentrations of 10, 20, 40, 80, and 160 μmol/L was added to the drug intervention group. Each group was provided with three holes, 10 μL of the MTT solution was added to each hole, and further incubation was carried out for 1 h after 12, 24, and 48 h, respectively. The absorbance (OD) value of each hole at 450 nm was measured using the microplate reader. The survival and inhibition rates of MH7A cells were calculated as follows: cell survival rate/%=(OD experimental hole-OD blank hole)/(OD control hole-OD blank hole) × 100%, cell inhibition rate/%=(OD control hole-OD experimental hole)/(OD control hole-OD blank hole) × 100%.

#### 2.9.5 RT-PCR detection

The mRNA expression of core genes was detected by SYBR Green I PCR Assay using the quantitative RT-PCR method. Total RNA from cells was extracted using the TRIzol reagent in the control group, positive drug group (80 μmol/L methotrexate), and drug intervention group (80 μmol/L *β-*sitosterol) and stored in an −80-°C refrigerator. Complementary DNA was synthesized from 1 μg RNA utilizing the SP6 High-Yield RNA Transcription Kit. The RT-PCR thermocycling condition contained initial denaturation at 55°C for 2 min and 95°C for 10 min, together with 50 cycles of 95°C for 15 s and 55°C for 1 min. Primers were synthesized by Shanghai Sangong Biotechnology Co., Ltd. (Table 2). The complementary DNA was amplified using specific primers. The expression difference was calculated using the 2^−ΔΔCT^ method and normalized to the loading control GAPDH.

**TABLE 2:** Primer sequences for RT-PCR.

Gene name	Forward (5′-3′)	Reverse (5′-3′)
ALB	TGTCACGGCGACCTGTTG	GGA​GAT​AGT​GGC​CTG​GTT​CTC​A
EGFR	CAG​AAG​CCA​TCT​CTG​ACT​CCC	GTC​CAG​TGG​TCA​ACA​AGG​TG
SRC	GAG​CGG​CTC​CAG​ATT​GTC​AA	CTG​GGG​ATG​TAG​CCT​GTC​TGT
HSP90AA1	TCG​CCT​TTC​AGG​CAG​AAA​TTG​C	CGA​CTT​TTG​TTC​CAC​GAC​CCA​TA
CASP3	CTT​GGC​GAA​ATT​CAA​AGG​ATG​G	CCC​GGG​TAA​GAA​TGT​GCA​TAA
MMP9	AGC​CCG​GGA​ATT​CGT​TTA​AAC​CTC​ACC​ATG​AGC​CCC​CTG​CAG	TTT​ATT​GCG​GCC​AGC​GGC​CGC​TCA​GAA​CAG​ATC​CAC​TAG​TTG​GGA​T

#### 2.9.6 Western blotting

Western blotting was utilized to detect the expression levels of anti-rheumarthritis-related proteins. A measure of 15 μL DMSO was added to the MH7A cells of the control group. A measure of 15 μL *β-*sitosterol at final concentrations of 20, 40, 80, and 160 μmol/L was added to MH7A cells of the drug intervention group, respectively. Then, MH7A cells were cultured for 48 h, the cells were placed on ice, and the total protein of each group of cells was extracted using the RIPA lysis buffer assay kit, followed by protein quantification using the BCA assay (Tiangen Biotech (Beijing) Co., Ltd., China). A measure of 20 μg aliquot of each protein sample was denatured by heating at 100°C for 10 min, separated by 10% SDS-PAGE, and transferred to polyvinylidene fluoride (PVDF) Immobilon membranes. The primary antibodies used for Western blotting were rabbit matrix metalloproteinase 9 (MMP9) (cat. no. AF5234), cysteine–aspartate protease 3 (CASP3) (cat. no. AF6370), heat shock protein 90 kDa alpha (cytosolic), class A member 1 (HSP90AA1) (cat. no. AF7140), steroid receptor coactivator (SRC) (cat. no. AF1831), epidermal growth factor receptor (EGFR) (cat. no. AF1330), and albumin (ALB) (cat. no. AF6183), and glyceraldehyde-3-phosphate dehydrogenase (GAPDH) (cat. no. AF1186) at a ratio of 1: 1,000. The secondary antibody used was goat anti-rabbit IgG (H+L) (cat. no. A0208) at a ratio of 1:1,000 (Beyotime Institute of Biotechnology, Shanghai, China). The corresponding primary antibody was added at room temperature for 1 h according to instructions and incubated overnight at 4°C; the secondary antibody was added after membrane washing and incubated in a shaking table for 2 h; and the membrane was washed with TBST three times, 10 min each time. An ECL imaging system was used for lambent development. The experiment was repeated a minimum of three times, and therefore, the value was measured and analyzed. The gray values of the bands were quantified using ImageJ 2.0 software (National Institutes of Health, https://imagej.net/software/fiji/downloads). The images of the original Western blots of proteins are provided in Supplementary Figure 4.

### 2.10 Statistical analysis

The raw experimental data were presented and analyzed using SPSS 20.0 software statistically. The quantitative data were finally expressed as the mean ± standard deviation. The groups were compared and analyzed using a one-way analysis of variance (ANOVA). Tukey’s *post hoc* test was carried out following the one-way ANOVA. The data were visualized using GraphPad Prism 9.0. The difference was statistically significant (*p < 0.05*).

## 3 Results

### 3.1 *β*-Sitosterol anti-RA component target screening results

**FIGURE 1:**
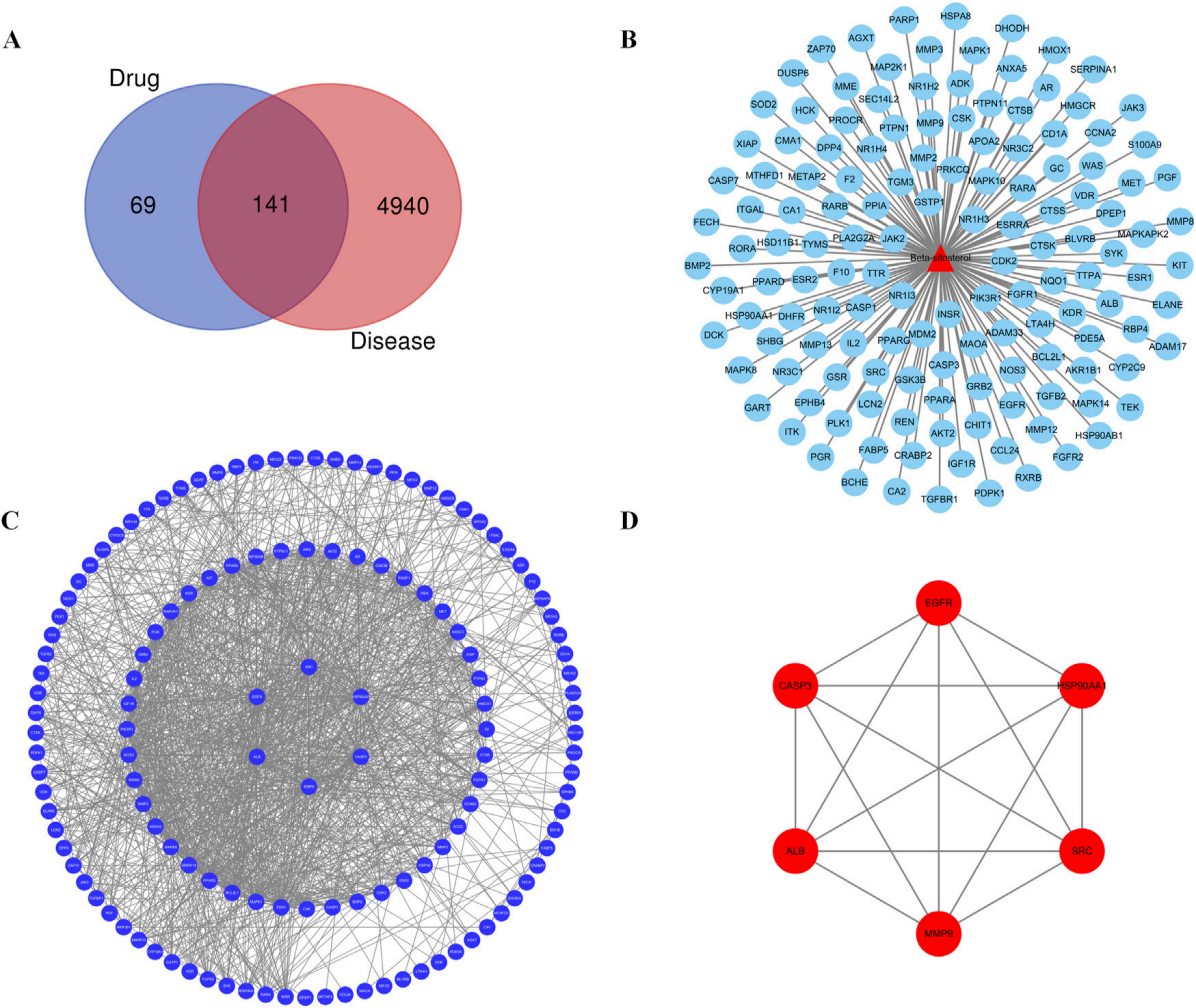
**(A)** Venn diagram of the common target gene screening of VR and RA-related targets. **(B)** Active ingredient *β*-sitosterol in the *VR* anti-RA target network. **(C)** PPI network of *β*-sitosterol in anti-RA. **(D)** Key PPI network of *β*-sitosterol in anti-RA.

Further maps of the targets were obtained from the disease and component databases and then normalized and standardized using the UniProt database for consistent naming. Invalid and duplicate targets were deleted, and a total of 210 effective targets for *β-*sitosterol were obtained. A total of 5,081 RA-related genes were retrieved. After the intersection, 141 common targets related to *β-*sitosterol and RA were finally obtained (Figure 1A).

### 3.2 Construction of the PPI network of common target genes

The common or core target genes of “component–disease” were imported into the STRING database, the biological species was set as “homosapiens,” the PPI network of common or core target genes was built, and finally, the construction results were imported into Cytoscape 3.7.2 software for visual presentation. The PPI networks of the “component–disease” common target or core target genes were presented in Figure 1B. The results of network topology analysis of 141 target genes showed that there are 30 core target genes with node degree values greater than the average value in the network, and the top 6 core target genes with different degree values, betweenness centrality, and closeness centrality are MMP9, CASP3, HSP90AA1, SRC, EGFR, and ALB, respectively. The topology analysis results are given in Figure 1C and Table 3. The PPI analysis of the first six core targets was carried out again, with the results shown in Figure 1D. It was shown that the degree values between the six core targets were high, and the relationship was also close. Therefore, these six targets were selected for further research.

**TABLE 3:** Corresponding core target genes of *β*-sitosterol based on the degree value.

Gene name	Degree	Betweenness centrality	Closeness centrality
ALB	87	0.16690869	0.74033149
EGFR	69	0.06553308	0.66336634
SRC	68	0.05405453	0.65365854
HSP90AA1	64	0.07096108	0.65048544
CASP3	60	0.0432541	0.64114833
MMP9	59	0.04297561	0.62325581
ESR1	57	0.03059738	0.61187215
MAPK1	52	0.02966793	0.59821429
PPARG	48	0.04722952	0.59555556
BCL2L1	48	0.01498664	0.59030837
MAPK14	46	0.0200853	0.5826087
ANXA5	44	0.01359018	0.58008658
MAPK8	44	0.01315706	0.5826087
MMP2	40	0.0108487	0.56302521
NOS3	40	0.0224873	0.5751073
MDM2	40	0.01934122	0.57758621
PIK3R1	39	0.00902607	0.53174603
GRB2	37	0.00635954	0.54032258
IL2	37	0.02857632	0.56302521
IGF1R	37	0.00426479	0.54918033
MAP2K1	34	0.0074821	0.56066946
PGR	34	0.00724636	0.54471545
PPARA	33	0.035052	0.55144033
KIT	33	0.01326571	0.54251012
KDR	33	0.00310502	0.54251012
PTPN11	32	0.00404951	0.53174603
HSP90AB1	32	0.00610305	0.55144033
AKT2	31	0.00497923	0.54918033
JAK2	31	0.00208704	0.54032258
GSK3B	30	0.00250847	0.54251012
AR	30	0.00299651	0.53386454
PARP1	28	0.00316685	0.536
REN	27	0.01287251	0.52964427
XIAP	27	0.00306453	0.51340996
NR3C1	27	0.01850575	0.53174603
MET	27	0.00132981	0.52140078
HMOX1	26	0.02358089	0.536
PTPN1	26	0.00305854	0.51145038
F2	24	0.0123031	0.52140078
SOD2	23	0.01094282	0.53174603

### 3.3 GO function enrichment analysis results

**FIGURE 2:**
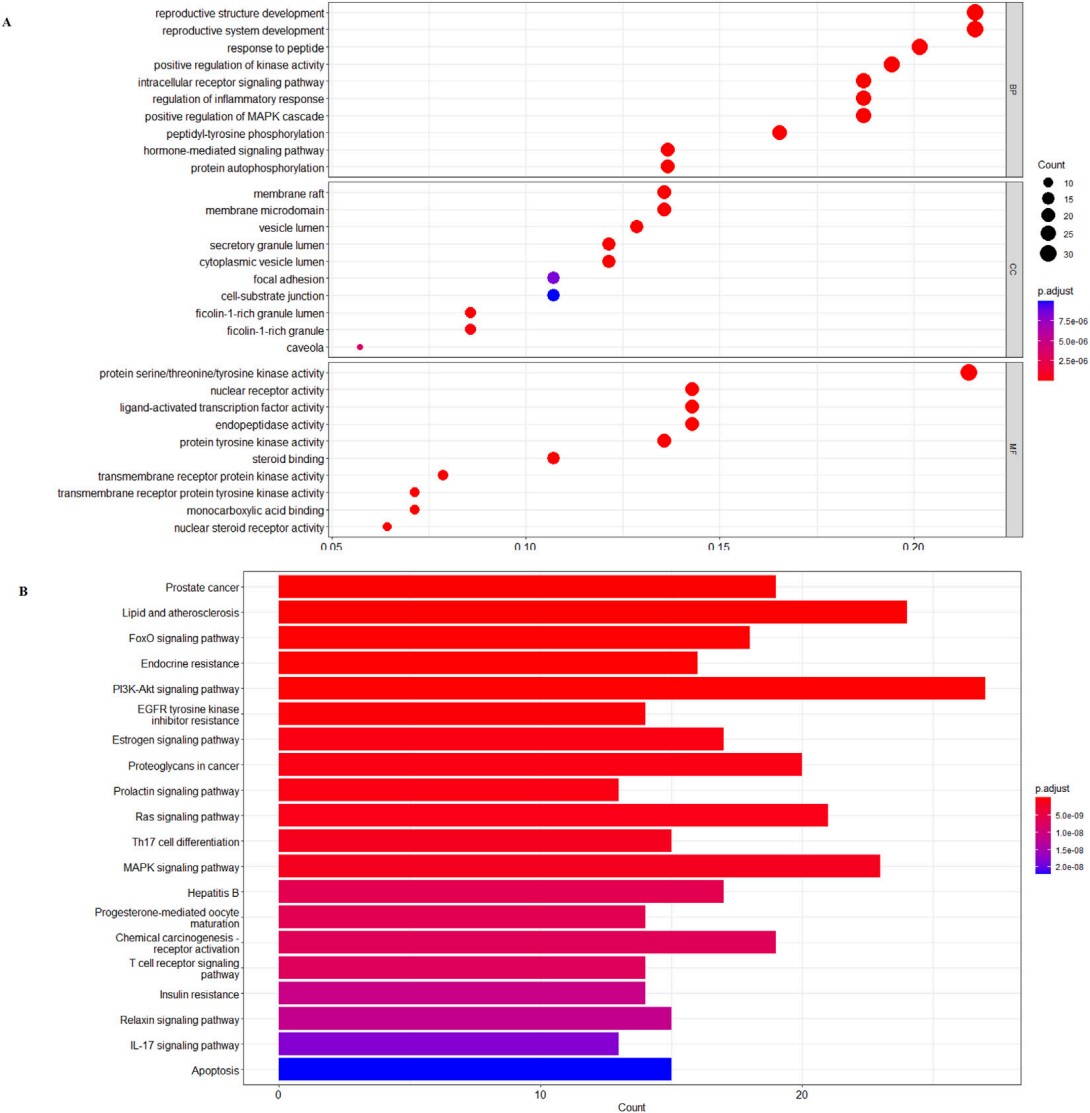
**(A)** *β*-sitosterol prevention and treatment of the RA gene GO enrichment analysis bubble diagram. **(B)** KEGG pathway enrichment histogram chart of the active ingredients of *β*-sitosterol in the treatment of RA.

R 4.0.4 was used to analyze the GO function annotation of 24 core targets, and a total of 285 entries of BP (*p < 0.05*), 270 entries of CC (*p < 0.05*), and 225 entries of MF (*p < 0.05*) were obtained. The top 20 items were selected for visual display according to the *p-*value, and the GO function annotation analysis bubble diagram (Figure 2A) was drawn. Among them, BP mainly involves reproductive structure development, reproductive system development, response to peptide, positive regulation of kinase activity, intracellular receptor signaling pathway, regulation of inflammatory response, positive regulation of the mitogen-activated protein kinase (MAPK) cascade, peptidyl–tyrosine phosphorylation, hormone-mediated signaling pathway, and protein autophosphorylation. CC mainly involves membrane raft, membrane microdomain, vesicle lumen, secretory granule lumen, cytoplasmic vesicle lumen, focal adhesion, cell–substrate junction, ficolin-1-rich granule lumen, ficolin-1-rich granule, and caveola. MF mainly involves protein serine/threonine/tyrosine kinase activity, nuclear receptor activity, ligand-activated transcription factor activity, endopeptidase activity, protein tyrosine kinase activity, steroid binding, transmembrane receptor protein kinase activity, transmembrane receptor protein tyrosine kinase activity, monocarboxylic acid binding, and nuclear steroid receptor activity.

### 3.4 Enrichment analysis of KEGG pathway results

**FIGURE 3:**
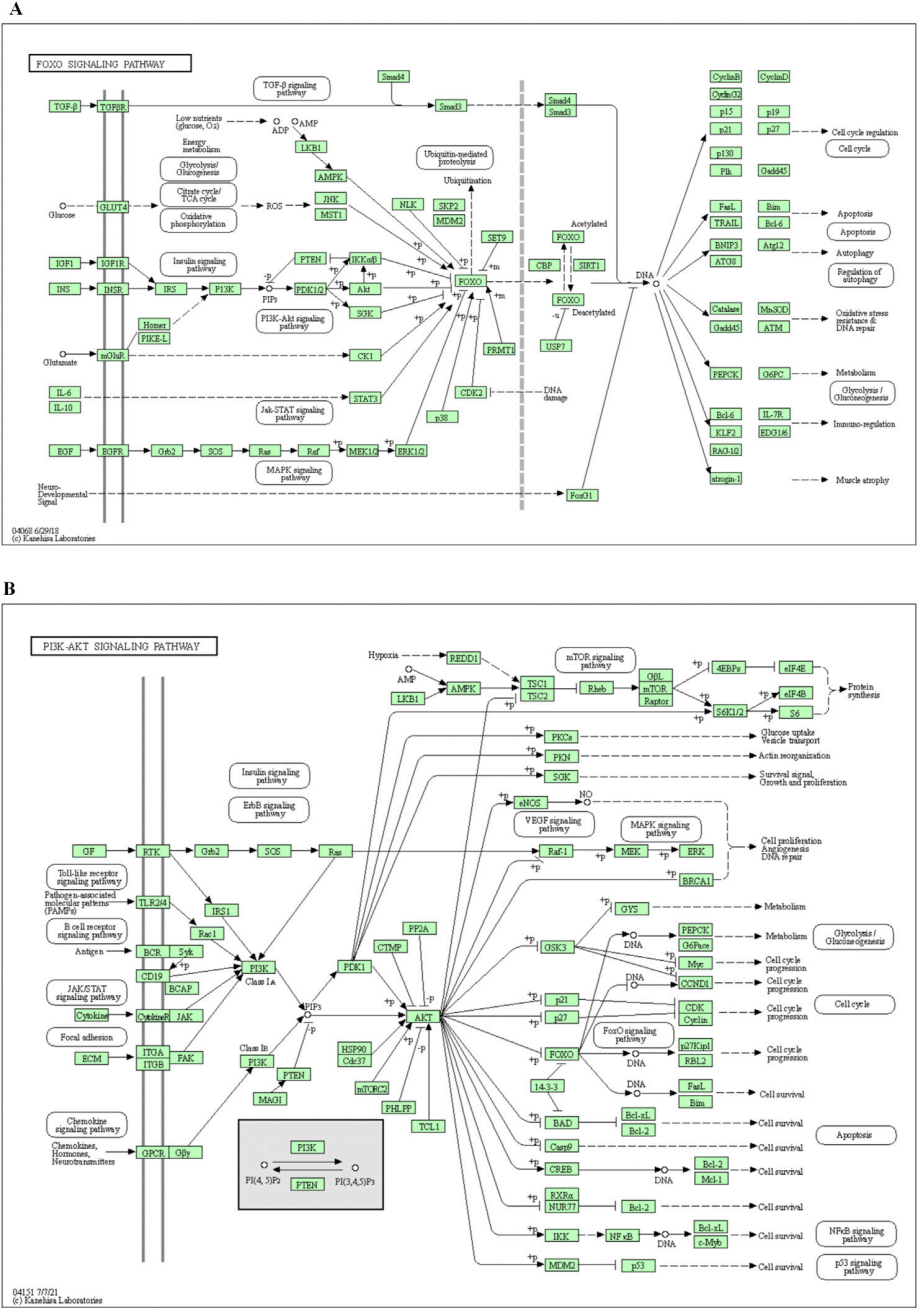
Annotated map of the target genes related to *β*-sitosterol in VR on RA-related signaling pathways. **(A)** FoxO signaling pathway. **(B)** PI3K/AKT signaling pathway.

R 4.0.4 software was used to conduct KEGG pathway enrichment analysis on the core target, 105 signal pathways (*p < 0.05*) were obtained, the top 20 channels were selected for visual processing, and the KEGG signal pathway histogram was drawn (Figure 2B). The rich factor represents the ratio of the number of genes in this pathway in the related genes to the total number of genes in this pathway in all annotation genes. The larger the value, the higher the enrichment degree. The analysis of the enriched pathways mainly included prostate cancer, lipid and atherosclerosis, FoxO signaling pathway, endocrine resistance, PI3K/AKT signaling pathway, EGFR tyrosine kinase inhibitor resistance, estrogen signaling pathway, proteoglycans in cancer, prolactin signaling pathway, Ras signaling pathway, Th17 cell differentiation, MAPK signaling pathway, hepatitis B, progesterone-mediated oocyte maturation, chemical carcinogenesis–receptor activation, T-cell receptor signaling pathway, insulin resistance, relaxin signaling pathway, IL-17 signaling pathway, and apoptosis pathways. An annotated map of the locations of key target genes of *β-*sitosterol in RA-related pathways is shown in Figure 3. It was revealed that most of the key target genes are associated with the FoxO signaling pathway (Figure 3A) and PI3K/AKT signaling pathway (Figure 3B), which may influence the therapeutic effect on RA.

### 3.5 Molecular docking results

**FIGURE 4:**
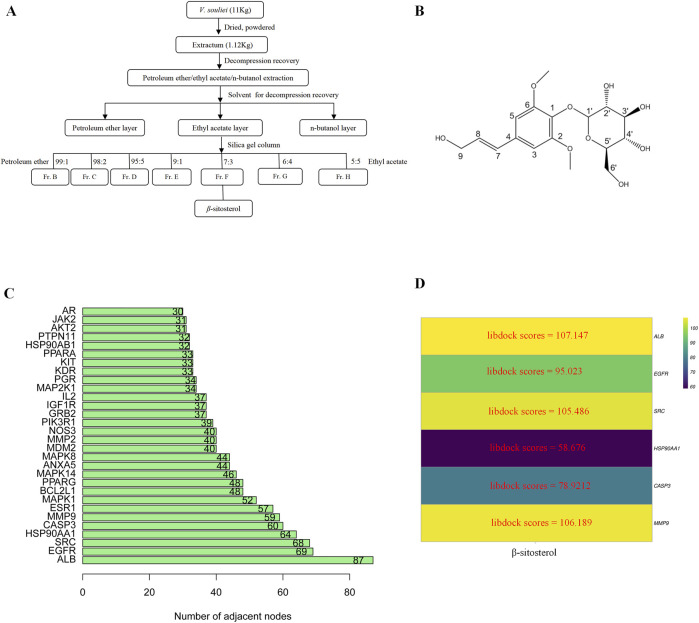
**(A)** Flowchart of the extraction of *β*-sitosterol from VR*.* **(B)** 2D structure of *β*-sitosterol. **(C)** Bar diagram of corresponding core target genes of *β*-sitosterol based on the degree value. **(D)** Heatmap of the main active ingredients of VR with six core target genes.

**FIGURE 5:**
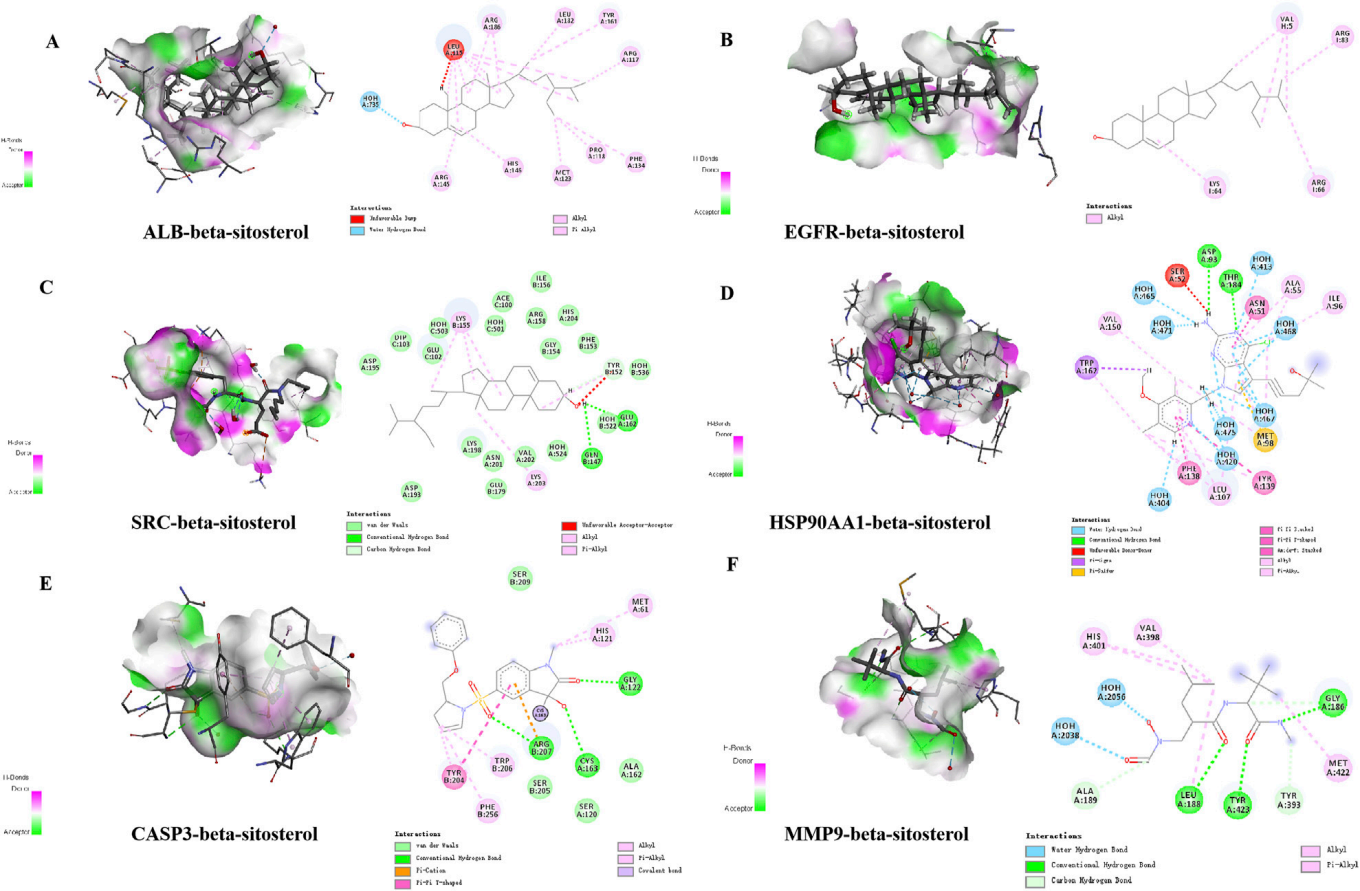
Detailed “drug–target” interactions of the molecular docking verification. **(A)** *β*-sitosterol–ALB, **(B)** *β*-sitosterol–EGFR, **(C)** *β*-sitosterol–SRC, **(D)** *β*-sitosterol–HSP90AA1, **(E)** *β*-sitosterol–CASP3, and **(F)** *β*-sitosterol–MMP9.

*β-*Sitosterol was derived from fraction F (7:3) of VR (Figure 4A). In addition, β-sitosterol is also an organic compound with the molecular formula C_29_H_50_O, which is a white crystalline solid (Figure 4B). According to the normal PPI network, the top six degree values were finally selected as the core targets for molecular docking with the compound of *β*-sitosterol (Figure 4C). The substance primarily interacts with the core targets via conventional hydrogen bonding, hydrocarbon bonding, and pi–pi conjugation mechanisms. Furthermore, the LibDock score of each core target with the compound of *β-*sitosterol is shown in Figure 4D. In addition, the 3D and 2D figures of *β-*sitosterol docking with core genes *ALB* (Figure 5A), *EGFR* (Figure 5B), *SRC* (Figure 5C), *HSP90AA1* (Figure 5D), *CASP3* (Figure 5E), and *MMP9* (Figure 5F) are presented. The LibDock score indicates the binding degree of the ligand and target protein crystal. The higher the LibDock score, the higher the predicted binding activity of small molecules to receptors. Among them, the LibDock score of *β-*sitosterol combined with ALB, EGFR, SRC, HSP90AA1, CASP3, and MMP9 was 106.189, 78.9212, 58.676, 105.486, 95.023, and 107.147, respectively (Table 4). It was revealed that *β-*sitosterol exhibits good binding with RA-related targets. Drug discovery is a complex process of *in vitro* testing, *in vivo* validation, and other steps for candidate drugs. Based on 3D structure, molecular docking experiments can predict the conformation and binding affinity of complexes. Molecular docking experiments only proved that *β-*sitosterol mainly affects the functional expression of proteins by binding to different binding sites through hydrogen bonding, hydrophobicity, van der Waals forces, and electrostatic forces. The LibDock score also shows whether *β-*sitosterol has good binding with RA-related targets. However, the specific functional changes in the protein still need to be verified based on specific experiments, and we also verified the protein’s function in anti-rheumatoid diseases through the PCR and WB test methods in subsequent experiments.

**TABLE 4:** Results of molecular docking.

Compound	Target	PDB	LibDock score
*β*-Sitosterol	ALB	7VR0	107.147
*β*-Sitosterol	EGFR	3P0V	95.023
*β*-Sitosterol	SRC	1A07	105.486
*β*-Sitosterol	HSP90AA1	7S9I	58.676
*β*-Sitosterol	CASP3	1GFW	78.9212
*β*-Sitosterol	MMP9	1GKC	106.189

### 3.6 Inhibitory effect of *β*-sitosterol on MH7A cell proliferation

**FIGURE 6:**
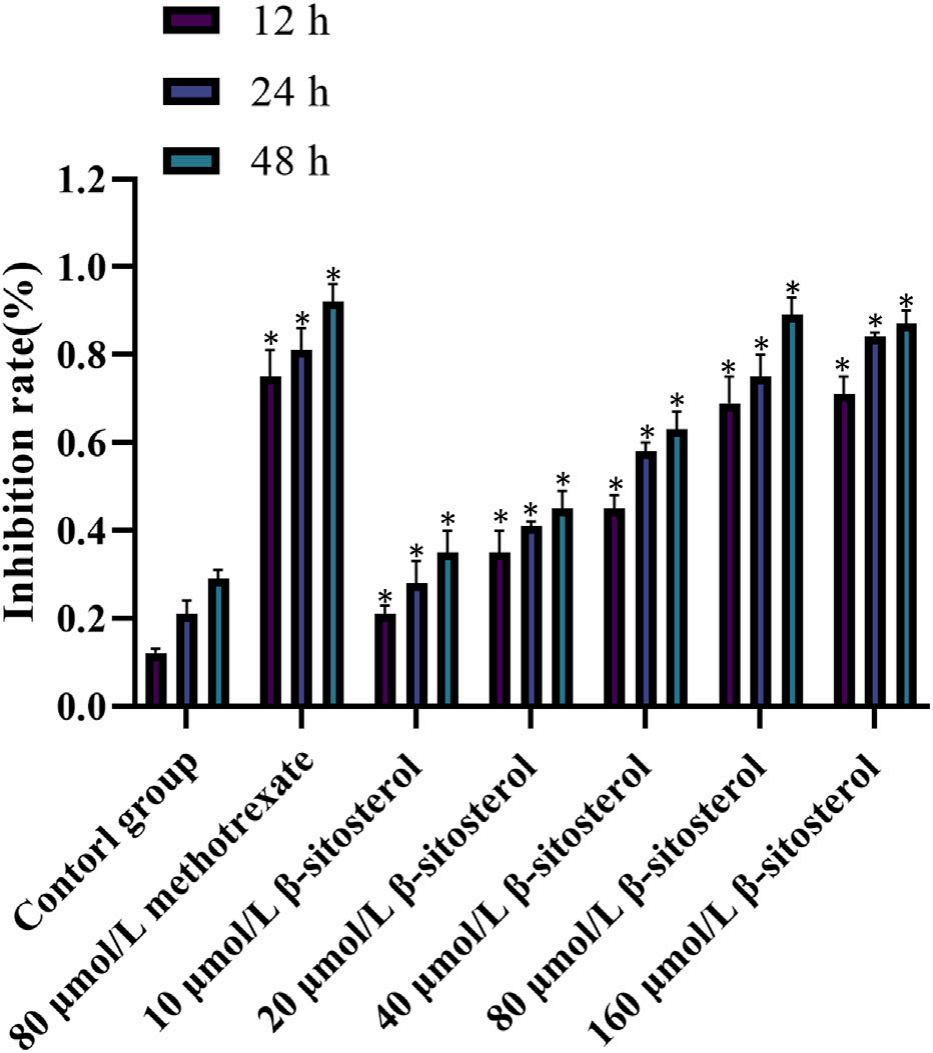
Cell inhibition rate of *β*-sitosterol on MH7A cells. The data represent the mean ± SD. ^*^ *p* < 0.05 vs. control group.

After cells were treated with different concentrations of *β-*sitosterol, the MTT method was used to detect the proliferation of cells at 12, 24, and 48 h. The results showed that the inhibition rate of *β-*sitosterol was significantly higher than that of the control group, and the difference was statistically significant (*p < 0.05*). With the increase in concentration and time, the inhibition of *β-*sitosterol was stronger at a concentration of 10 μmol/L to 160 μmol/L at 12 h and 24 h, respectively. In addition, the inhibition of β-sitosterol was stronger at the concentration of 10 μmol/L to 40 μmol/L at 48 h. Interestingly, it is apparent that 80 μmol/L *β-*sitosterol exhibits stronger inhibition than 160 μmol/L *β-*sitosterol at 48 h (Figure 6).

### 3.7 Results of RT-PCR

**FIGURE 7:**
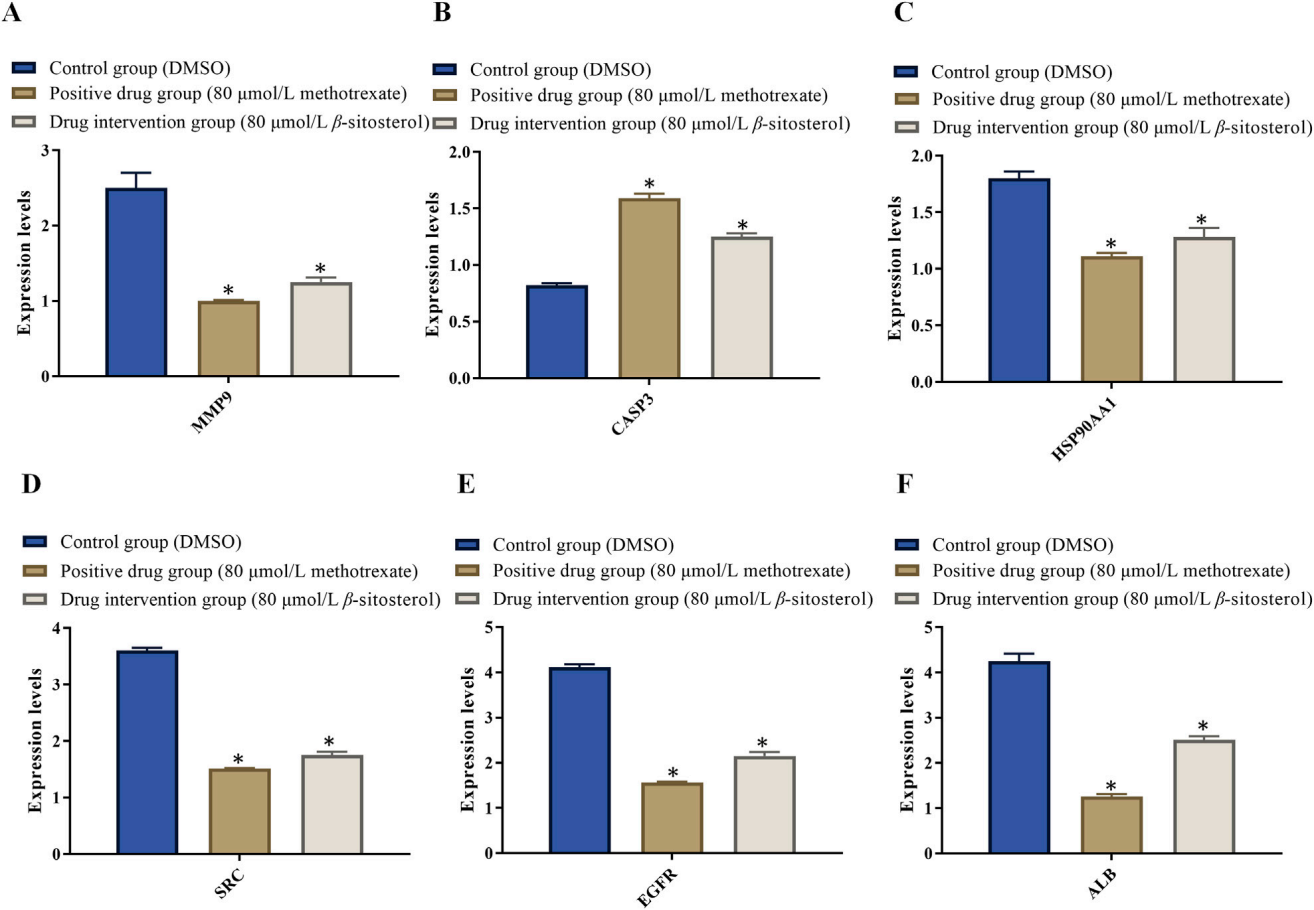
Effect of *β*-sitosterol on the mRNA expression of related genes of RA. **(A)** MMP9, **(B)** CASP3, **(C)** HSP90AA1, **(D)** SRC, **(E)** EGFR, and **(F)** ALB. The data represent the mean ± SD. ^*^ *p* < 0.05 vs. control group.

The doses of 80 μmol/L *β-*sitosterol exhibit a good effect on anti-rheumarthritis in our experiment. To maintain the same concentration, we use 80 μmol/L methotrexate for further research. On one hand, the rationale for the choice of doses 80 μmol/L *β-*sitosterol and 80 μmol/L methotrexate is based on the result of MTT and the expression of related proteins. On the other hand, the rationale for the choice of these doses/(concentration range) and the treatment time are based on the literature (Zhu et al., 2023; Ju et al., 2004; Yingzhan et al., 2023; Zhang et al., 2022; Awad et al., 2007). Therefore, MH7A cells were finally treated with DMSO (control group), 80 μmol/L methotrexate (positive drug group), and 80 μmol/L *β-*sitosterol (drug intervention group) for 24 h, respectively. In the control group, RT-PCR results showed that the relative mRNA expression levels of MMP9, HSP90AA1, SRC, EGFR, and ALB significantly increased, while CASP3 significantly reduced, indicating that inflammation occurred. Compared with the control group, the relative mRNA expression levels of MMP9 (Figure 7A), HSP90AA1 (Figure 7B), SRC (Figure 7C), EGFR (Figure 7D), and ALB (Figure 7E) significantly decreased, while CASP3 (Figure 7F) increased in the 80 μmol/L *β-*sitosterol group and 80 μmol/L methotrexate group (*p < 0.05*) (Figure 7). Encouragingly, these findings suggested that 80 μmol/L *β-*sitosterol plays an anti-rheumarthritis role by regulating the transcription and translation of multiple key molecules during the occurrence and development of rheumarthritis.

### 3.8 Effect of *β*-sitosterol on the expression of rheumarthritis-related proteins

**FIGURE 8:**
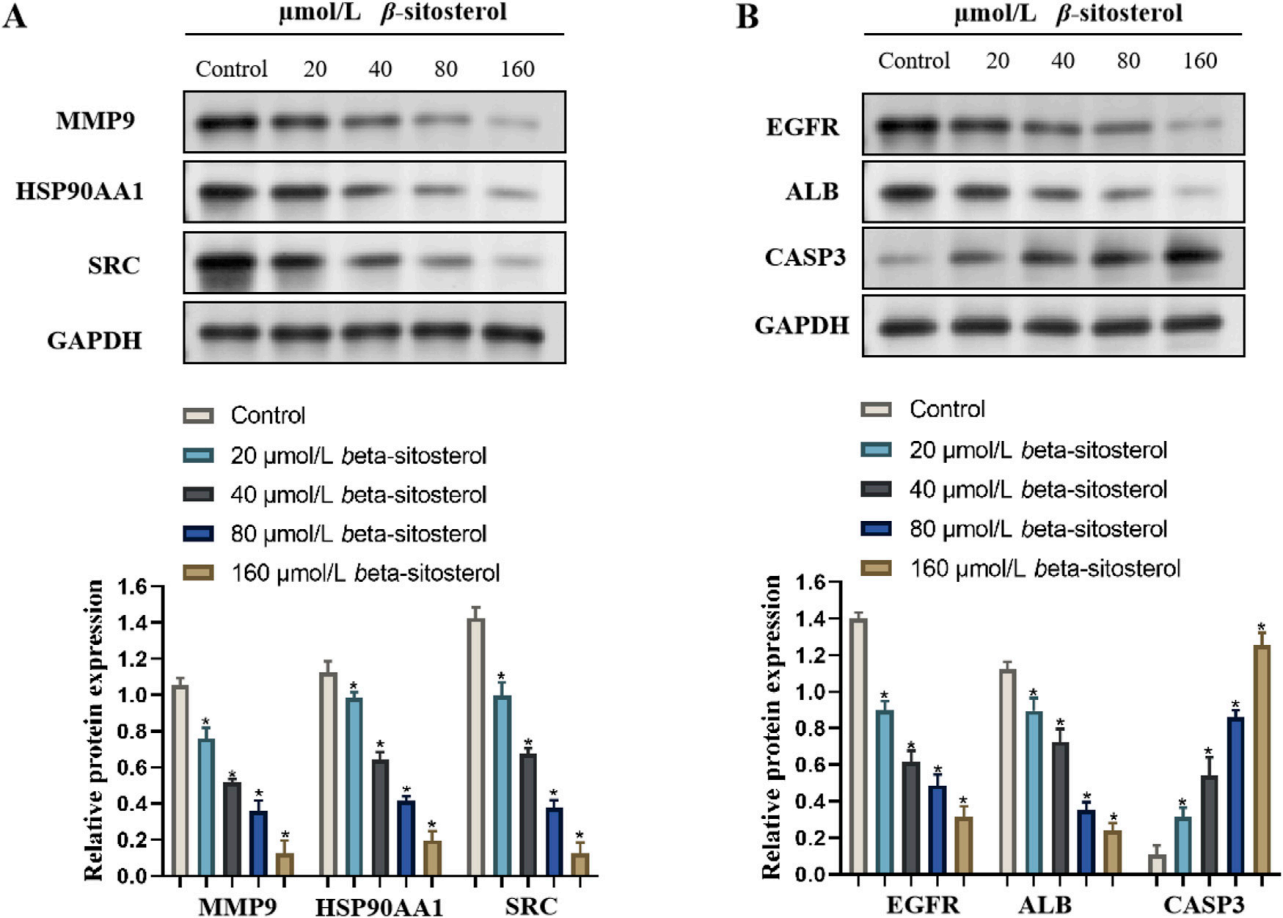
Western blotting bands of *β*-sitosterol on the related protein expressions **(A)** MMP9, HSP90AA1, SRC, **(B)** EGFR, ALB, and CASP3 and the quantification data of RA.

To explore the anti-rheumarthritis mechanism of *β-*sitosterol, the protein expression levels of MMP9, HSP90AA1, SRC, EGFR, ALB, and CASP3 were finally detected in the present study. Compared with the control group, *β-*sitosterol significantly decreased the expression of MMP9, HSP90AA1, SRC, EGFR, and ALB and significantly increased the expression of CASP3 proteins at concentrations of 20–160 μmol/L (*p < 0.05*) (Figure 8). The findings confirmed that *β-*sitosterol exerts an anti-rheumarthritis effect by inhibiting MMP9, HSP90AA1, SRC, EGFR, and ALB and promoting CASP3.

## 4 Discussion

RA is an autoimmune disease characterized by synovitis, cartilage destruction, and bone erosion. It has a high incidence rate, high recurrence rate, and high disability rate, affecting approximately 0.5%–1% of the global population (Finckh, 2019; Min et al., 2021). Currently, the pathogenesis of RA is still unclear. Previous research suggests that osteoclasts, T cells, inflammatory cytokines, signal pathways, and other factors may play an important role in the occurrence and development of RA (Hairul-Islam et al., 2017). Modern medicine attributes the pathogenic risk factors of RA to congenital genetics, the surrounding environment, and endocrine factors (Kobayashi et al., 2008). The exact pathogenesis of RA is quite complex, but abnormal immune responses are considered an important trigger of autoimmune diseases. Currently, the concept of T-cell-mediated adaptive immunity as a driving force for the transition stage of autoimmune diseases has been widely validated. In T cells, the pro-inflammatory effects of helper T (Th) 17 cells and the repair function of regulatory T cells (Treg) are closely related to autoimmune diseases, especially RA (Moon et al., 2013). In addition to macrophages, T cells and their respective secreted cytokines play a crucial role in the process of RA allergy, and an increasing number of studies have shown that fibroblast-like synoviocytes (FLSs) play a more important role in participating in joint injury (Bustamante et al., 2017). Therefore, MH7A joint FLSs are a commonly used cell for studying RA.

The treatment drugs mainly include anti-rheumatic drugs, non-steroidal anti-inflammatory drugs, and glucocorticoids to control the disease, but these drugs need to be used for a long time, and some exhibit resistant properties (Haraoui, 2009). Therefore, searching for new anti-RA drugs is one of the issues that researchers need to solve urgently. In the early stage, several basic components of VR were separated and extracted by the research group. *β-*Sitosterol is one of the main components derived from VR, which has had significant effects on RA in recent years (Liu et al., 2019; Qian et al., 2022). The present study revealed that *β*-sitosterol may interact with 164 potential anti-RA targets. GO and KEGG analyses of potential targets showed that the related pathways of the anti-RA mechanism of *β*-sitosterol were closely related to the FoxO signaling pathway and PI3K/AKT signaling pathway, respectively. The PPI analysis showed that MMP9, CASP3, HSP90AA1, SRC, EGFR, ALB, and other potential targets play an instance role in anti-RA. The purpose of this study was to figure out the mechanism of *β-*sitosterol that may reduce the inflammatory reaction and delay the progress of RA disease.

MMP9 plays an important role in physiological and pathological processes such as immune inflammation, cell migration, proliferation, and apoptosis. In the inflammatory state of the body, immune cells recruited to the microcirculation adhere to the surface of endothelial cells by secreting inflammatory factors and vascular cell adhesion molecule (VCAM) 1. This induces endothelial cells to express MMP9, promotes the migration of immune inflammatory cells across endothelial cells, and, at the same time, upregulates the activity of MMP9 (Iyer et al., 2016). Some studies have shown that the expression of MMP9 in serum and osteoarticular cartilage is increased in patients with RA (Li et al., 2013). In addition, MMP9 can participate in bone development, remodeling, and repair. During osteoclast differentiation, the expression of MMP9 increases and stimulates bone absorption (Kim et al., 2019). The occurrence and development of RA disease are also closely related to the process of apoptosis. The caspase family protease is a key enzyme in apoptosis signal transduction, and CASP3 can participate in initiating apoptosis (Shrestha and Clark, 2021). The tumor necrosis factor (TNF) inhibits the expression of tumor necrosis factor receptor (TNFR)-1 and CASP3 on the cell membrane and promotes the proliferation of FLSs in RA (Ni et al., 2019). A significant feature of arthritis is cartilage degradation, and CASP3, as a core factor affecting the process of apoptosis, may play a role in both internal and external apoptosis pathways. For disease prevention and treatment, inhibiting the expression of CASP3 is essential to reduce chondrocyte apoptosis (Hua et al., 2020). This result may apply to the treatment effect for RA. Administrated with *β-*sitosterol, the expression of CASP3 proteins was significantly increased, which is similar to previous research results (Nakayama et al., 2012; Ding et al., 2019). Research has reported that HSP90AA1 is related to the process of regulating cell differentiation (Zuehlke et al., 2015), and the transcription factor nuclear factor kappa-B (NF-κB) is related to the regulation of the bone cell life process and can perform its transcription function by inducing the expression of HSP90AA1 (Hu et al., 2020). ALB levels are considered clinical indicators reflecting malnutrition and chronic diseases. Extensive research revealed that the low level of albumin reduced the immune level of the body, and the anti-infection ability was weak. Therefore, ALB is one of the indicators of the nutritional status of the body and also an obvious sign of systemic immune and inflammatory response (Kalantar-Zadeh et al., 2005). It was also found that the ALB-to-fibrinogen is a useful biomarker to predict systemic inflammation, especially in RA (Yang et al., 2018). SRC may act as a key target of vascular endothelial growth factor (VEGF) and hypoxia-inducible factor (HIF)-1 signaling pathways (Guo et al., 2020). In addition, TNF-α can be inhibited by inhibiting the SRC/FAK/ERK1/2 and AKT signaling pathways, thus regulating the expression level of inflammatory factors in RA (Kumar et al., 2016). EGFR is a receptor for epithelial growth factor (EGF) cell proliferation and signal transduction. The EGFR signaling pathway plays an important role in cell growth, proliferation, differentiation, and other physiological processes (Fraguas et al., 2011). A previous study reported that EGFR and its ligands may be involved in the pathogenesis of RA (Yuan et al., 2013). In addition, it was revealed that RA is associated with rs17337023 single-nucleotide polymorphism (SNP) in the EGFR gene and increased the serum level of the EGFR protein, which may be a therapeutic target for RA (Huang et al., 2017).

Designing drugs targeting specific molecular target proteins is an important means of achieving precision medicine (Manzari et al., 2021). By delving into the structure and function of disease-related target proteins, researchers can develop drugs with higher selectivity and fewer side effects (Evans and McLeod, 2003). Targeted therapy is a treatment method that targets specific molecular proteins to treat diseases by interfering with or regulating the function of these target proteins (Lee et al., 2018). The abovementioned study is basically consistent with the target prediction of this study, indicating the characteristics of *β-*sitosterol in treating RA through multiple targets. In order to better study the effect of *β-*sitosterol on RA, molecular docking verification was further carried out. The results showed that *β-*sitosterol, as a potential active ingredient of VR, has protective and therapeutic effects on RA, and its mechanism may be related to reducing the generation of inflammatory factors, inhibiting the activation of enzymes, anti-oxidation, improving the ability to eliminate free radicals, immune regulation, and other ways (Bin Sayeed et al., 2016). Each molecule or protein exhibits different feedback by binding with *β-*sitosterol. In the present study, *β-*sitosterol at concentrations of 80 and 160 μmol/L significantly decreased the expression of MMP9, HSP90AA1, SRC, EGFR, and ALB while significantly reducing CASP3 proteins compared with the control group (*p < 0.05*). Therefore, we believed that those molecules may all exhibit positive feedback by binding with *β-*sitosterol. The cell experiment results showed that the inhibition rate of MH7A cells was significantly higher in *β-*sitosterol of the drug intervention group than that of the control group with the increase in concentration and time, thus indicating that *β-*sitosterol may help inhibit the activity of MH7A cells. The limitation of the research mainly includes the lack of animal experiment validation; it only focuses on cell experiment validation. In addition, the targets of pathways related to RA are not fully evaluated. The results of the study highlight the need to use a more representative sample for future research.

## 5 Conclusion

In conclusion, *β-*sitosterol exhibits anti-RA activity *via* multiple targets (MMP9, CASP3, HSP90AA1, SRC, EGFR, and ALB) and signaling pathways (FoxO and PI3K/AKT pathways). Network pharmacology and cell-based experimental verification predict and confirm that *β-*sitosterol may be used to treat RA through anti-proliferation and anti-inflammatory mechanisms. These findings provide a theoretical basis for the further application of *β-*sitosterol in the treatment of RA-related diseases.

## Data Availability

The datasets presented in this study can be found in online repositories. The names of the repository/repositories and accession number(s) can be found in the article/Supplementary Material.

## References

[B1] Awad A. B. Chinnam M. F. C. S. Fink C. S. Bradford P. G. (2007). beta-Sitosterol activates Fas signaling in human breast cancer cells. Phytomedicine 14 (11), 747–754. 10.1016/j.phymed.2007.01.003 17350814

[B2] Bin Sayeed M. S. Karim S. M. R. Sharmin T. Morshed M. M. (2016). Critical analysis on characterization, systemic effect, and therapeutic potential of beta-sitosterol: a plant-derived orphan phytosterol. Medicines 3 (4), 29. 10.3390/medicines3040029 28930139 PMC5456237

[B3] Bouic P. J. (2001). The role of phytosterols and phytosterolins in immune modulation: a review of the past 10 years. Curr. Opin. Clin. Nutr. and Metabolic Care 4 (6), 471–475. 10.1097/00075197-200111000-00001 11706278

[B4] Bustamante M. F. Garcia-Carbonell R. Whisenant K. D. Guma M. (2017). Fibroblast-like synoviocyte metabolism in the pathogenesis of rheumatoid arthritis. Arthritis Res. and Ther. 19, 110–112. 10.1186/s13075-017-1303-3 28569176 PMC5452638

[B5] Chemin K. Klareskog L. Malmström V. (2016). Is rheumatoid arthritis an autoimmune disease? Curr. Opin. rheumatology 28 (2), 181–188. 10.1097/BOR.0000000000000253 26780425

[B6] Cheng D. Guo Z. Zhang S. (2015). Effect of *β*-sitosterol on the expression of HPV E6 and p53 in cervical carcinoma cells. Contemp. Oncology/Współczesna Onkol. 19 (1), 36–42. 10.5114/wo.2015.50011 PMC4507893 26199569

[B7] Ding Y. Zhao Q. Wang L. (2019). Pro-apoptotic and anti-inflammatory effects of araloside A on human rheumatoid arthritis fibroblast-like synoviocytes. Chemico-biological Interact. 306, 131–137. 10.1016/j.cbi.2019.04.025 31004595

[B8] Evans W. E. McLeod H. L. (2003). Pharmacogenomics—drug disposition, drug targets, and side effects. N. Engl. J. Med. 348 (6), 538–549. 10.1056/NEJMra020526 12571262

[B9] Finckh A. (2019). JAK inhibitors in the management of rheumatoid arthritis. Rev. Medicale Suisse 15 (641), 528–532. 30860322

[B10] Fraguas S. Barberán S. Cebrià F. (2011). EGFR signaling regulates cell proliferation, differentiation and morphogenesis during planarian regeneration and homeostasis. Dev. Biol. 354 (1), 87–101. 10.1016/j.ydbio.2011.03.023 21458439

[B11] Guo X. Ji J. Jose Kumar Sreena G. S. Hou X. Luo Y. Fu X. (2020). Computational prediction of antiangiogenesis synergistic mechanisms of total saponins of panax japonicus against rheumatoid arthritis. Front. Pharmacol. 11, 566129. 10.3389/fphar.2020.566129 33324204 PMC7723436

[B12] Gupta E. (2020). β-Sitosterol: predominant phytosterol of therapeutic potential. Innovations food Technol., 465–477. 10.1007/978-981-15-6121-4_32

[B13] Hairul-Islam M. I. Saravanan S. Thirugnanasambantham K. Chellappandian M. Raj C. S. D. Karikalan K. (2017). Swertiamarin, a natural steroid, prevent bone erosion by modulating RANKL/RANK/OPG signaling. Int. Immunopharmacol. 53, 114–124. 10.1016/j.intimp.2017.10.022 29078090

[B14] Haraoui B. (2009). Assessment and management of rheumatoid arthritis. J. Rheumatology Suppl. 82, 2–10. 10.3899/jrheum.090124 19509324

[B15] Hu B. Zhang S. Liu W. Wang P. Chen S. Lv X. (2020). Inhibiting heat shock protein 90 protects nucleus pulposus-derived stem/progenitor cells from compression-induced necroptosis and apoptosis. Front. cell Dev. Biol. 8, 685. 10.3389/fcell.2020.00685 32850811 PMC7427414

[B16] Hua L. Wang F. Q. Du H. W. Fan J. Wang Y. F. Wang L. Q. (2020). Upregulation of caspase-3 by high glucose in chondrocyte involves the cytoskeleton aggregation. Eur. Rev. Med. Pharmacol. Sci. 24 (11), 5925–5932. 10.26355/eurrev_202006_21485 32572905

[B17] Huang C. M. Chen H. H. Chen D. C. Huang Y. C. Liu S. P. Lin Y. J. (2017). Rheumatoid arthritis is associated with rs17337023 polymorphism and increased serum level of the EGFR protein. PLoS One 12 (7), e0180604. 10.1371/journal.pone.0180604 28700691 PMC5507450

[B18] Huang Y. Gong Z. Yan C. Zheng K. Zhang L. Li J. (2023). Investigation on the mechanisms of *Zanthoxylum bungeanum* for treating diabetes mellitus based on network pharmacology, molecular docking, and experiment verification. BioMed Res. Int. 2023, 9298728. 10.1155/2023/9298728 36874926 PMC9977524

[B19] Iyer R. P. Jung M. Lindsey M. L. (2016). MMP-9 signaling in the left ventricle following myocardial infarction. Am. J. Physiology-Heart Circulatory Physiology 311 (1), H190–H198. 10.1152/ajpheart.00243.2016 PMC4967202 27208160

[B20] Jiang G. Sun C. Wang X. Mei J. Li C. Zhan H. (2022). Hepatoprotective mechanism of Silybum marianum on nonalcoholic fatty liver disease based on network pharmacology and experimental verification. Bioengineered 13 (3), 5216–5235. 10.1080/21655979.2022.2037374 35170400 PMC8974060

[B21] Ju Y. H. Clausen L. M. Allred K. F. Almada A. L. Helferich W. G. (2004). *β*-sitosterol, *β*-sitosterol glucoside, and a mixture of *β*-sitosterol and *β*-sitosterol glucoside modulate the growth of estrogen-responsive breast cancer cells *in vitro* and in ovariectomized athymic mice. J. Nutr. 134 (5), 1145–1151. 10.1093/jn/134.5.1145 15113961

[B22] Kalantar-Zadeh K. Kilpatrick R. D. Kuwae N. McAllister C. J. Alcorn Jr H. Kopple J. D. (2005). Revisiting mortality predictability of serum albumin in the dialysis population: time dependency, longitudinal changes and population-attributable fraction. Nephrol. Dial. Transplant. 20 (9), 1880–1888. 10.1093/ndt/gfh941 15956056

[B23] Kim Y. Kim J. Lee H. Shin W. R. Lee S. Lee J. (2019). Tetracycline analogs inhibit osteoclast differentiation by suppressing MMP-9-mediated histone H3 cleavage. Int. J. Mol. Sci. 20 (16), 4038. 10.3390/ijms20164038 31430857 PMC6719029

[B24] Kobayashi S. Momohara S. Kamatani N. Okamoto H. (2008). Molecular aspects of rheumatoid arthritis: role of environmental factors. FEBS J. 275 (18), 4456–4462. 10.1111/j.1742-4658.2008.06581.x 18662304

[B25] Kumar A. Sunita P. Jha S. Pattanayak S. P. (2016). Daphnetin inhibits TNF‐α and VEGF‐induced angiogenesis through inhibition of the IKK s/IκBα/NF‐κB, Src/FAK/ERK 1/2 and Akt signalling pathways. Clin. Exp. Pharmacol. Physiology 43 (10), 939–950. 10.1111/1440-1681.12608 27297262

[B26] Lee Y. T. Tan Y. J. Oon C. E. (2018). Molecular targeted therapy: treating cancer with specificity. Eur. J. Pharmacol. 834, 188–196. 10.1016/j.ejphar.2018.07.034 30031797

[B27] Li G. Zhang Y. Qian Y. Zhang H. Guo S. Sunagawa M. (2013). Interleukin-17A promotes rheumatoid arthritis synoviocytes migration and invasion under hypoxia by increasing MMP2 and MMP9 expression through NF-κB/HIF-1α pathway. Mol. Immunol. 53 (3), 227–236. 10.1016/j.molimm.2012.08.018 22960198

[B28] Liao P. C. Lai M. H. Hsu K. P. Kuo Y. H. Chen J. Tsai M. C. (2018). Identification of *β*-sitosterol as *in vitro* anti-inflammatory constituent in Moringa oleifera. J. Agric. food Chem. 66 (41), 10748–10759. 10.1021/acs.jafc.8b04555 30280897

[B29] Liu R. Hao D. Xu W. Li J. Li X. Shen D. (2019). β-Sitosterol modulates macrophage polarization and attenuates rheumatoid inflammation in mice. Pharm. Biol. 57 (1), 161–168. 10.1080/13880209.2019.1577461 30905278 PMC6442231

[B30] Mandal S. K. Debnath U. Kumar A. Thomas S. Mandal S. C. Choudhury M. D. (2020). Natural sesquiterpene lactones in the prevention and treatment of inflammatory disorders and cancer: a systematic study of this emerging therapeutic approach based on chemical and pharmacological aspect. Lett. Drug Des. and Discov. 17 (9), 1102–1116. 10.2174/1570180817999200421144007

[B31] Manzari M. T. Shamay Y. Kiguchi H. Rosen N. Scaltriti M. Heller D. A. (2021). Targeted drug delivery strategies for precision medicines. Nat. Rev. Mater. 6 (4), 351–370. 10.1038/s41578-020-00269-6 34950512 PMC8691416

[B32] Mao J. Yi M. Tao Y. Huang Y. Chen M. (2019). Costunolide isolated from Vladimiria souliei inhibits the proliferation and induces the apoptosis of HepG2 cells. Mol. Med. Rep. 19 (2), 1372–1379. 10.3892/mmr.2018.9736 30569137

[B33] Mao J. Yi M. Wang R. Huang Y. Chen M. (2018). Protective effects of costunolide against D-galactosamine and lipopolysaccharide-induced acute liver injury in mice. Front. Pharmacol. 9, 1469. 10.3389/fphar.2018.01469 30618760 PMC6307542

[B34] Mao J. Zhan H. Meng F. Wang G. Huang D. Liao Z. (2022). Costunolide protects against alcohol‐induced liver injury by regulating gut microbiota, oxidative stress and attenuating inflammation *in vivo* and *in vitro* . Phytotherapy Res. 36 (3), 1268–1283. 10.1002/ptr.7383 35084790

[B35] Min H. K. Kim S. Lee J. Y. Kim K. W. Lee S. H. Kim H. R. (2021). IL-18 binding protein suppresses IL-17-induced osteoclastogenesis and rectifies type 17 helper T cell/regulatory T cell imbalance in rheumatoid arthritis. J. Transl. Med. 19 (1), 392–399. 10.1186/s12967-021-03071-2 34530864 PMC8444577

[B36] Moon S. J. Park J. S. Heo Y. J. Kang C. M. Kim E. K. Lim M. (2013). *In vivo* action of IL-27: reciprocal regulation of Th17 and Treg cells in collagen-induced arthritis. Exp. and Mol. Med. 45 (10), e46. 10.1038/emm.2013.89 24091748 PMC3809362

[B37] Moran-Moguel M. C. Petarra-del Rio S. Mayorquin-Galvan E. E. Zavala-Cerna M. G. (2018). Rheumatoid arthritis and miRNAs: a critical review through a functional view. J. Immunol. Res. 2018, 2474529. 10.1155/2018/2474529 29785401 PMC5896204

[B38] Nakayama H. Yaguchi T. Yoshiya S. Nishizaki T. (2012). Resveratrol induces apoptosis MH7A human rheumatoid arthritis synovial cells in a sirtuin 1-dependent manner. Rheumatol. Int. 32, 151–157. 10.1007/s00296-010-1598-8 20697895 PMC3253293

[B39] National Pharmacopoeia Committee (2020). Pharmacopoeia of the people’s Republic of China. Part 1, 50–51.

[B40] Ni S. Li C. Xu N. Liu X. Wang W. Chen W. (2019). Follistatin‐like protein 1 induction of matrix metalloproteinase 1, 3 and 13 gene expression in rheumatoid arthritis synoviocytes requires MAPK, JAK/STAT3 and NF‐κB pathways. J. Cell. physiology 234 (1), 454–463. 10.1002/jcp.26580 29932210

[B41] Novotny L. Abdel-Hamid M. E. Hunakova L. (2017). Anticancer potential of *β*-sitosterol. Int. J. Clin. Pharmacol. Pharmacother. 2 (129), 10–15344. 10.15344/2456-3501/2017/129

[B42] Qian K. Zheng X. X. Wang C. Huang W. G. Liu X. B. Xu S. D. (2022). *β*-sitosterol inhibits rheumatoid synovial angiogenesis through suppressing VEGF signaling pathway. Insights Exp. Pharmacol. Drug Discov. 2021 12. 10.3389/fphar.2021.816477 PMC8918576 35295740

[B43] Rashed K. (2020). Beta-sitosterol medicinal properties: a review article. Int. J. Sci. Invent. Today 9 (4), 208–212.

[B44] Rein P. Mueller R. B. (2017). Treatment with biologicals in rheumatoid arthritis: an overview. Rheumatology Ther. 4 (2), 247–261. 10.1007/s40744-017-0073-3 PMC5696285 28831712

[B45] Shi C. Liu J. Wu F. Zhu X. Yew D. T. Xu J. (2011). β-sitosterol inhibits high cholesterol-induced platelet *β*-amyloid release. J. bioenergetics Biomembr. 43 (6), 691–697. 10.1007/s10863-011-9383-2 21969169

[B46] Shrestha S. Clark A. C. (2021). Evolution of the folding landscape of effector caspases. J. Biol. Chem. 297, 101249. 10.1016/j.jbc.2021.101249 34592312 PMC8628267

[B47] Suzuki A. Yamamoto K. (2015). From genetics to functional insights into rheumatoid arthritis. Clin. Exp. Rheumatol. 33 (4 Suppl. 92), S40–S43. 26457422

[B48] Van Tuyl L. H. Lems W. F. Boers M. (2014). Measurement of stiffness in patients with rheumatoid arthritis in low disease activity or remission: a systematic review. BMC Musculoskelet. Disord. 15 (1), 28–36. 10.1186/1471-2474-15-28 24476506 PMC3914735

[B49] Verstappen S. M. M. Bijlsma J. W. J. Verkleij H. Buskens E. Blaauw A. A. M. Ter Borg E. J. (2004). Overview of work disability in rheumatoid arthritis patients as observed in cross‐sectional and longitudinal surveys. Arthritis Care and Res. 51 (3), 488–497. 10.1002/art.20419 15188338

[B50] Vivancos M. Moreno J. J. (2005). beta-Sitosterol modulates antioxidant enzyme response in RAW 264.7 macrophages. Free Radic. Biol. Med. 39 (1), 91–97. 10.1016/j.freeradbiomed.2005.02.025 15925281

[B51] Wang S. Du Q. Sun J. Geng S. Zhang Y. (2022). Investigation of the mechanism of Isobavachalcone in treating rheumatoid arthritis through a combination strategy of network pharmacology and experimental verification. J. Ethnopharmacol. 294, 115342. 10.1016/j.jep.2022.115342 35525528

[B52] Xu X. Luo H. Chen Q. Wang Z. Chen X. Li X. (2022). Detecting potential mechanism of vitamin D in treating rheumatoid arthritis based on network pharmacology and molecular docking. Front. Pharmacol. 13, 1047061. 10.3389/fphar.2022.1047061 36532774 PMC9749856

[B53] Yang W. M. Zhang W. H. Ying H. Q. Xu Y. M. Zhang J. Min Q. H. (2018). Two new inflammatory markers associated with disease activity score-28 in patients with rheumatoid arthritis: albumin to fibrinogen ratio and C-reactive protein to albumin ratio. Int. Immunopharmacol. 62, 293–298. 10.1016/j.intimp.2018.07.007 30048859

[B54] Yingzhan T. A. N. G. Jingyi Y. U. Wen Z. H. A. O. Juyan L. I. U. Hongying P. E. N. G. Zhang H. (2023). Total glucosides of *Rhizoma Smilacis Glabrae*: a therapeutic approach for psoriasis by regulating Th17/Treg balance. Chin. J. Nat. Med. 21 (8), 589–598. 10.1016/S1875-5364(23)60413-3 37611977

[B55] Yuan F. L. Li X. Lu W. G. Sun J. M. Jiang D. L. Xu R. S. (2013). Epidermal growth factor receptor (EGFR) as a therapeutic target in rheumatoid arthritis. Clin. Rheumatol. 32 (3), 289–292. 10.1007/s10067-012-2119-9 23179003

[B56] Zhang J. Wang R. Liang X. Bai H. T. Li Y. L. Sun S. (2022). Computation and molecular pharmacology to trace the anti-rheumatoid activity of *Angelicae Pubescentis Radix* . BMC Complementary Med. Ther. 22 (1), 312. 10.1186/s12906-022-03769-w PMC9701395 36435778

[B57] Zhou H. Y. Tang W. Jiang J. (2016). Effects of *β*-sitosterol and stigmasterol on non-alcoholic fatty liver disease *in vitro* . Acta Nutr. Sin. 38 (5), 456–461.

[B58] Zhu X. Li Y. Wang X. Huang Y. Mao J. (2023). Investigation of the mechanism of *Prunella vulgaris* in treatment of papillary thyroid carcinoma based on network pharmacology integrated molecular docking and experimental verification. Medicine 102 (17), e33360. 10.1097/MD.0000000000033360 37115092 PMC10145964

[B59] Zuehlke A. D. Beebe K. Neckers L. Prince T. (2015). Regulation and function of the human HSP90AA1 gene. Gene 570 (1), 8–16. 10.1016/j.gene.2015.06.018 26071189 PMC4519370

[B60] Zuo J. Ma S. (2024). Mechanism of beta-sitosterol on hypertrophic scar fibroblasts: an analysis based on network pharmacology. Chin. J. Tissue Eng. Res. 28 (2), 216.

