# Supplementary material for: Systematic pharmacology-based strategy to investigate the mechanism of beta-sitosterol for the treatment of rheumarthritis
Source: Front Genet. 2024 Dec 4;15:1507606. doi: 10.3389/fgene.2024.1507606 (PMC11652534; doi:10.3389/fgene.2024.1507606)
Supplement: Supplementary file 1 [file Table1.docx]

**
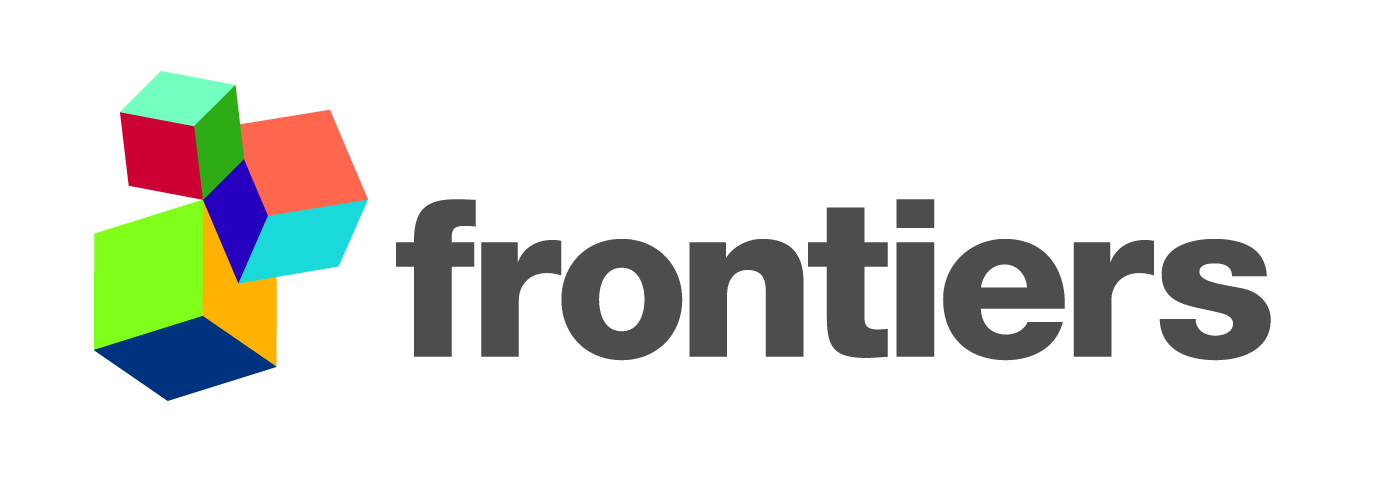
**

**Systematic pharmacology-based strategy to investigate the mechanism of beta-sitosterol derives from *Vladimiriae Radix* for the treatment of rheumarthritis**

**Xiaodong Wang^1^, Jingxin Mao^1,2*^**

^1^ Chongqing Medical and Pharmaceutical College, Chongqing 400030, China.

^2^ College of Pharmaceutical Sciences, Southwest University, Chongqing 400715, China.

**^*^Corresponding author:** Research Scientist and Dr. Jingxin Mao

**Email**: 2230040@cqmpc.edu.cn or [mmm518@163.com](mailto:ly20031079@163.com)

**Tel:** +86 13752922258

**Address**: Chongqing Medical and Pharmaceutical College, No. 82, Middle University Town Road, Shapingba District, Chongqing 400030, China.

**Running title: Mechanism of *β*-sitosterol on RA**

**List of supporting information**

Figure S1. The ^1^H-NMR of *β*-sitosterol.

Figure S2. The ^13^C-NMR of *β*-sitosterol.

Figure S3. The GC-MS result of *β*-sitosterol.

Figure S4. The images of the original western blots of proteins.


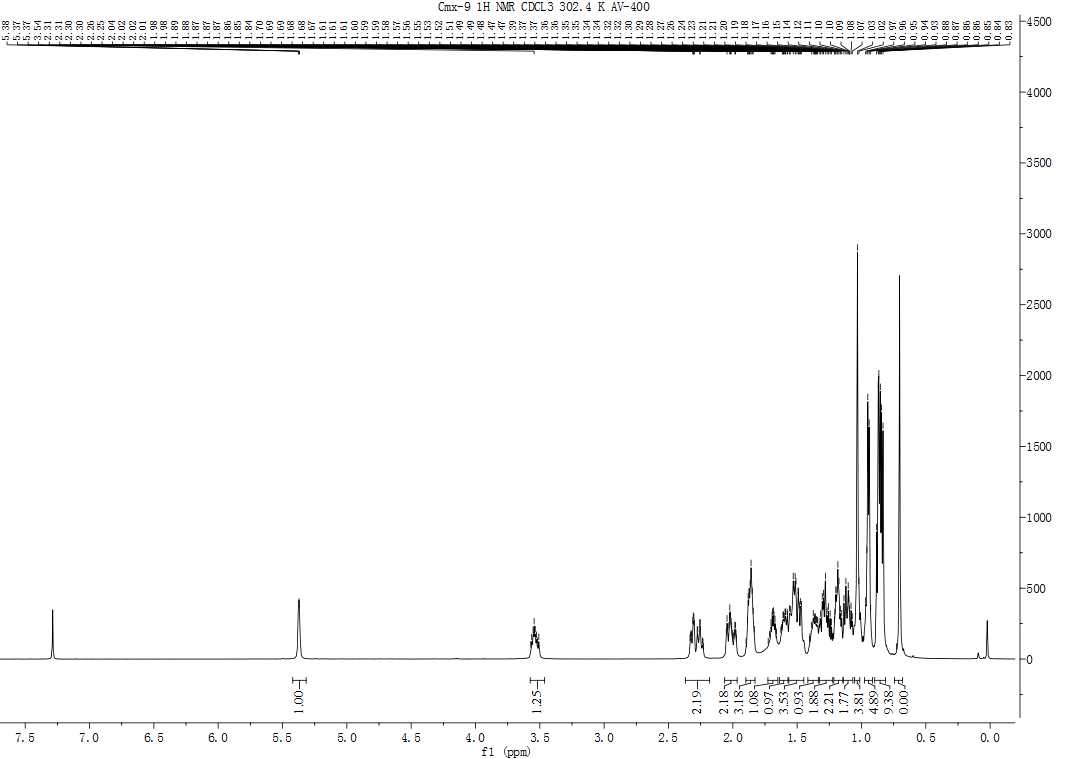


Figure S1. The ^1^H-NMR spectra of *β*-sitosterol.


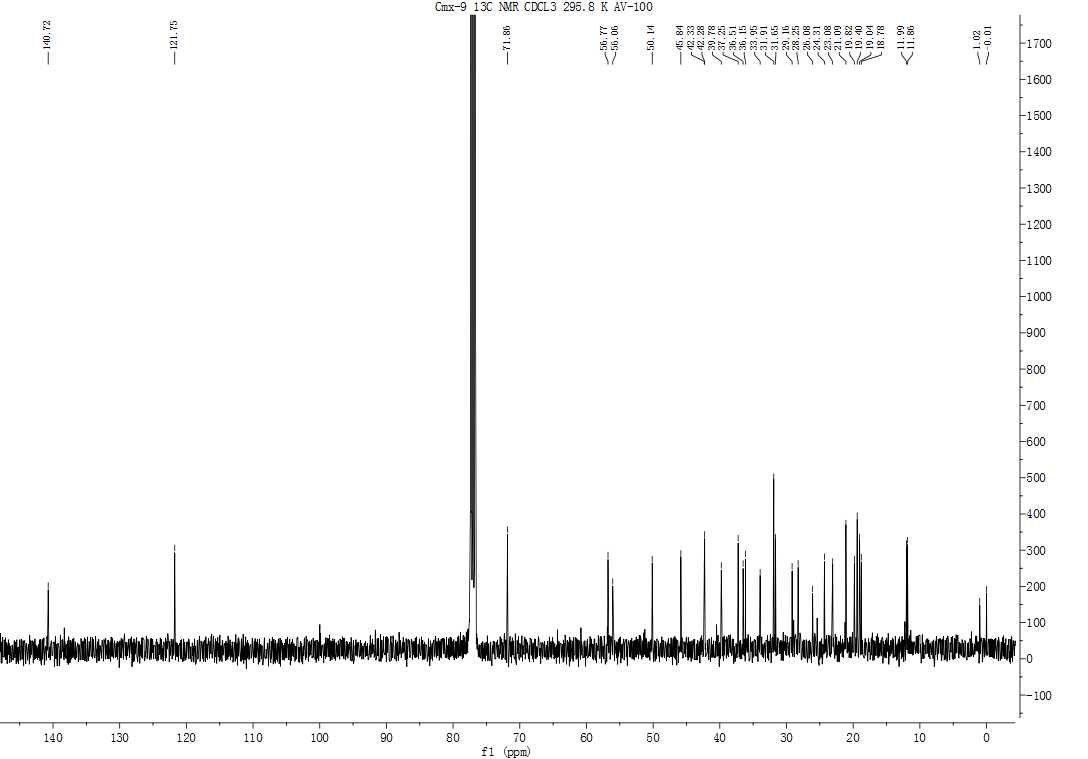


Figure S2. The ^13^C-NMR spectra of *β*-sitosterol.


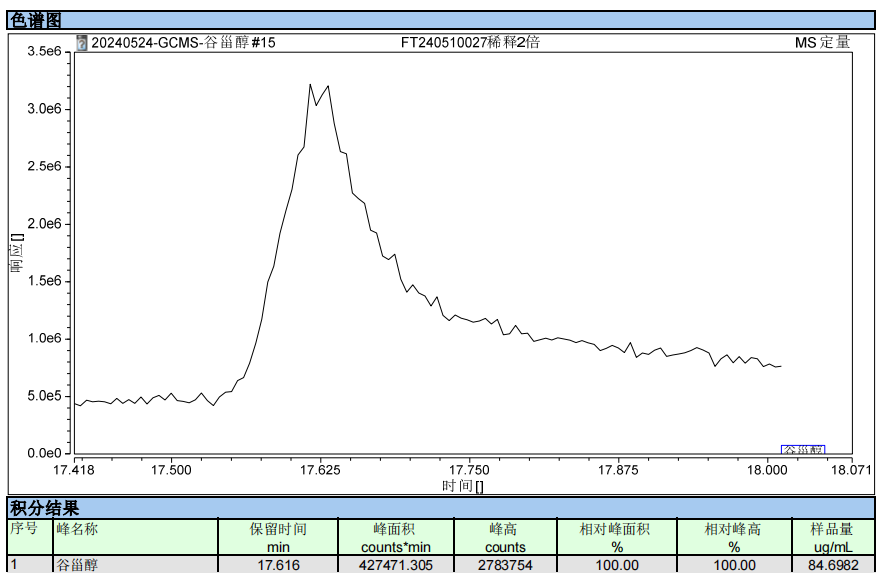


Figure S3. The GC-MS result of *β*-sitosterol.

| Proteins | Repeat 1 | Repeat 2 | Repeat 3 |
| --- | --- | --- | --- |
| HSP90AA1  (90 kDa) | 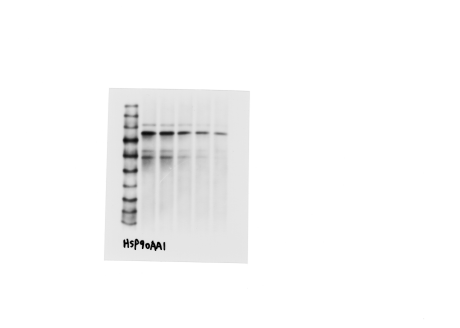 | 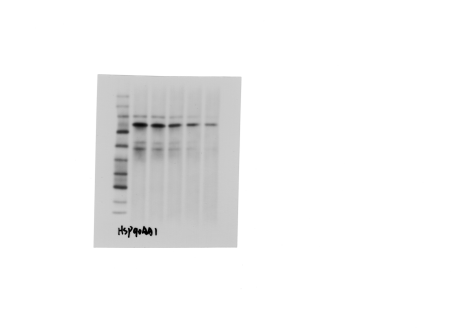 | 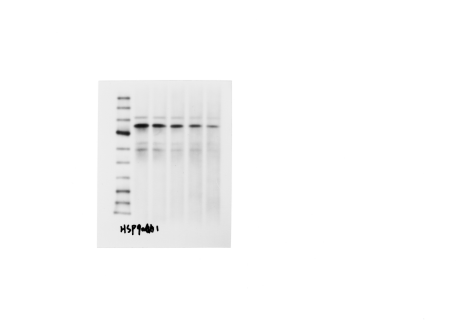 |
| MMP9  (82 kDa) | 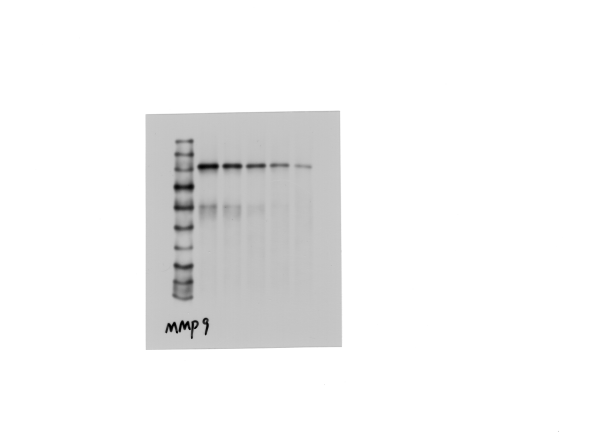 | 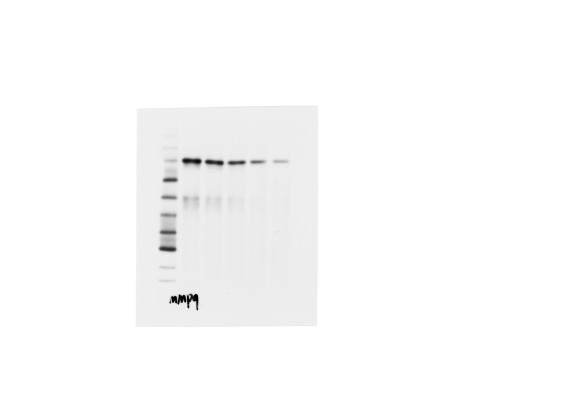 | 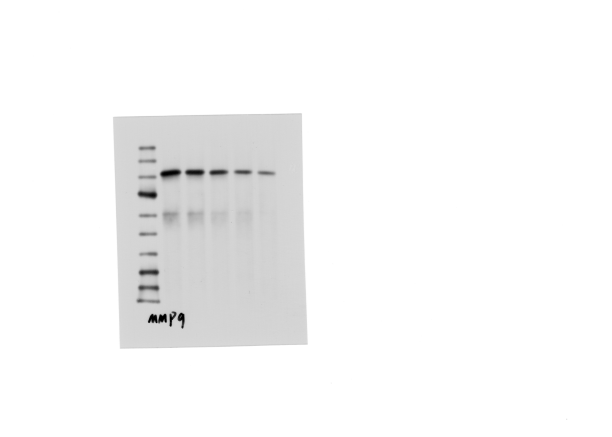 |
| SRC  (60 kDa) | 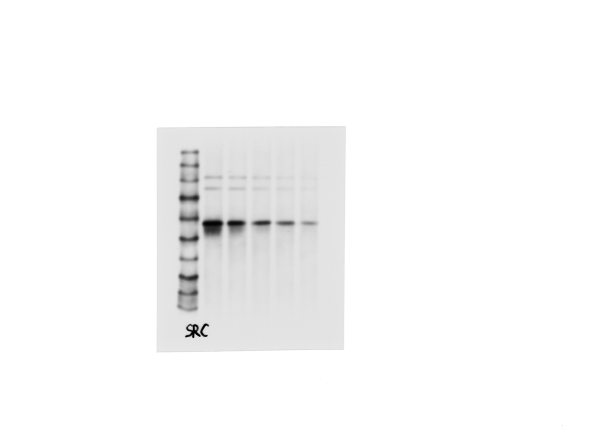 | 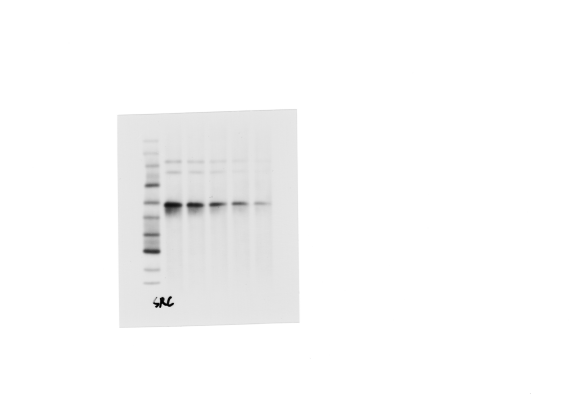 | 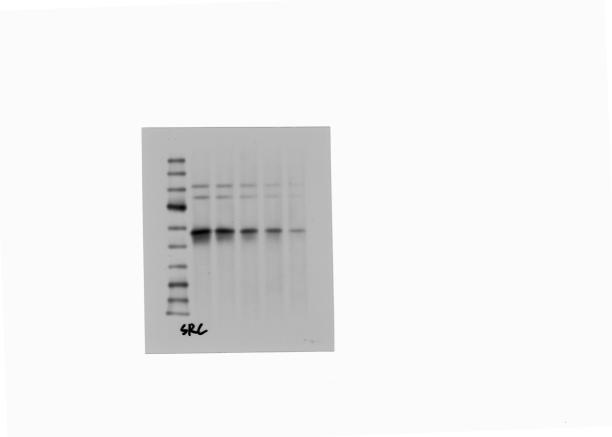 |
| GAPDH  (36 kDa) | 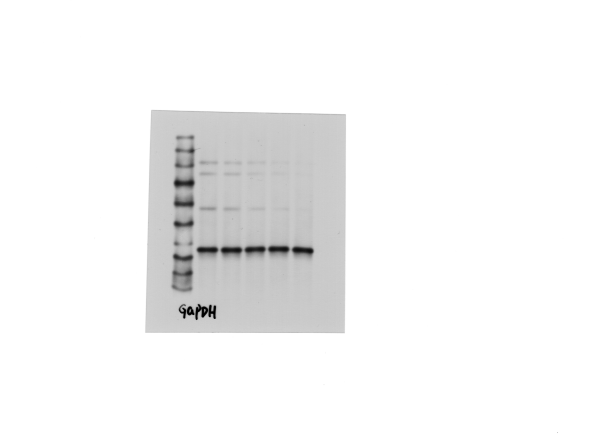 | 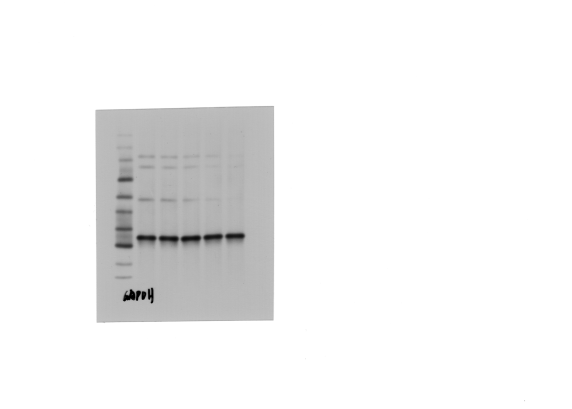 | 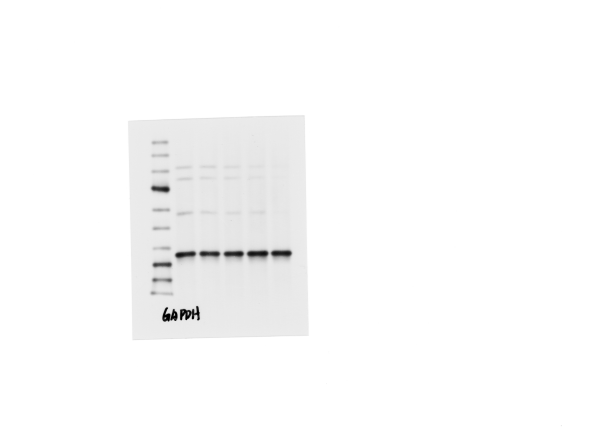 |
| ALB  (66.5 kDa) | 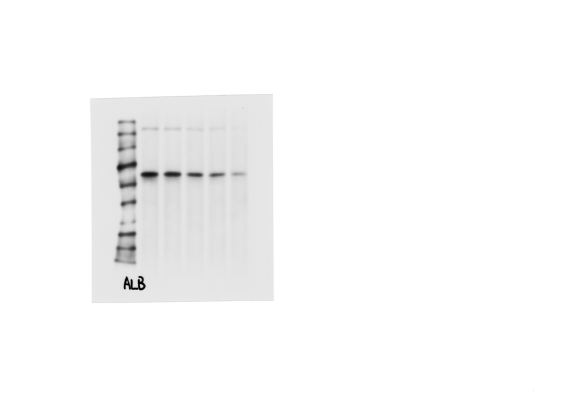 | 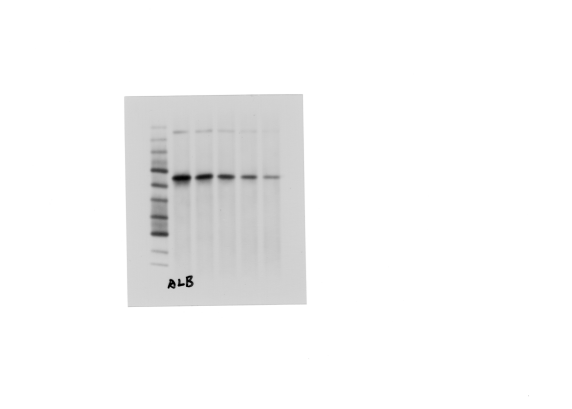 | 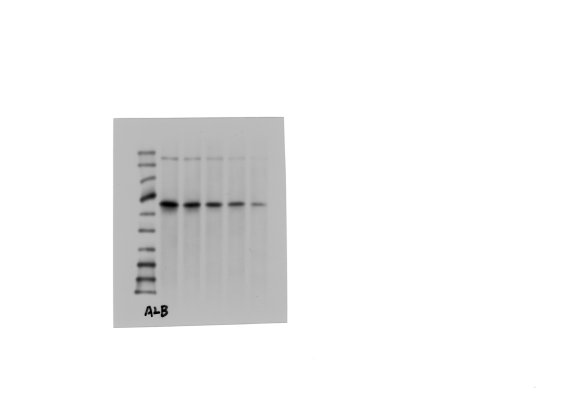 |
| CASP3  (35 kDa) | 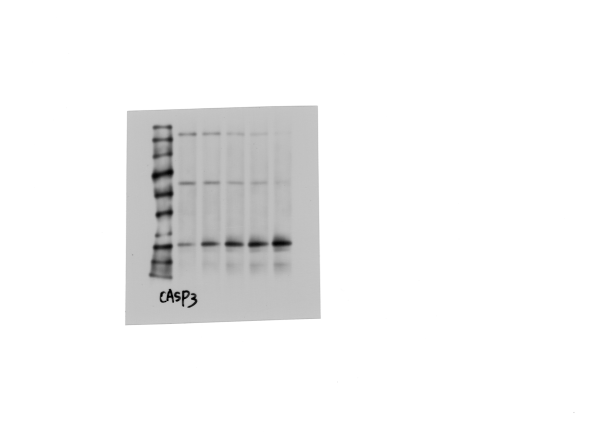 | 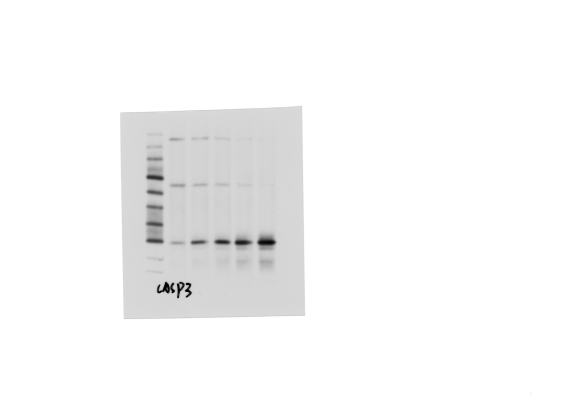 | 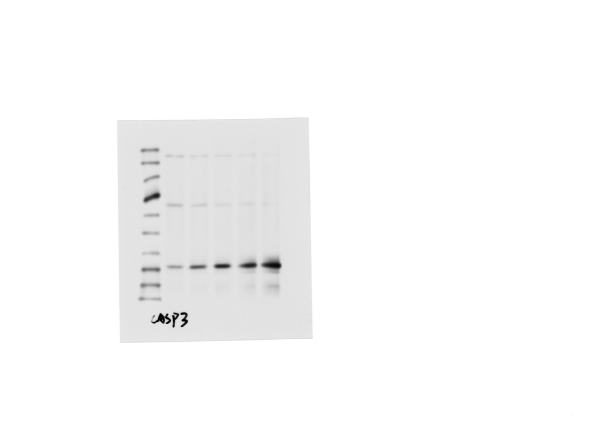 |
| EGFR  (152 kDa) | 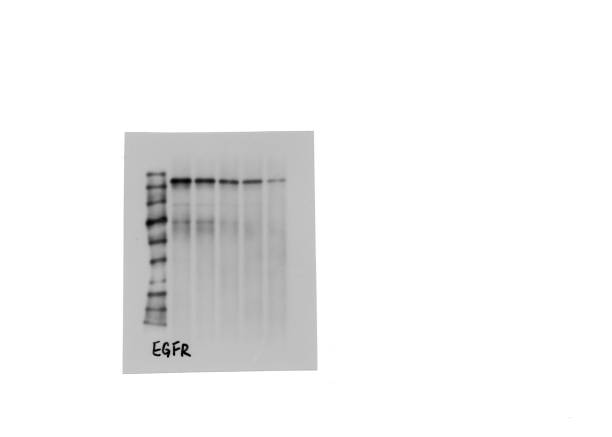 | 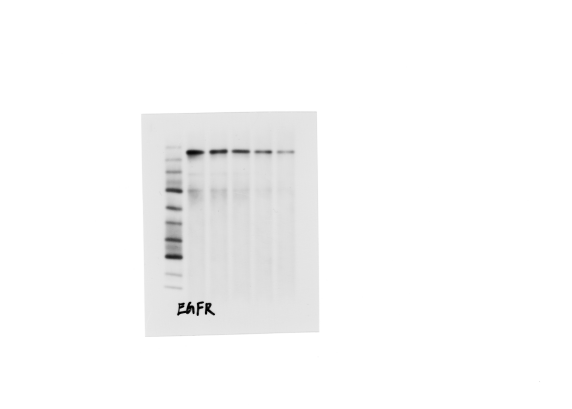 | 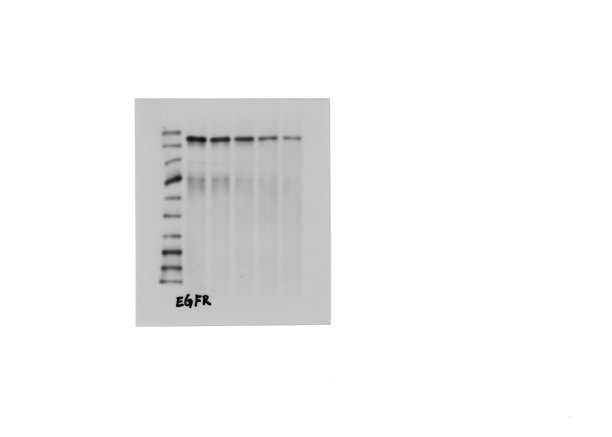 |
| GAPDH  (36 kDa) | 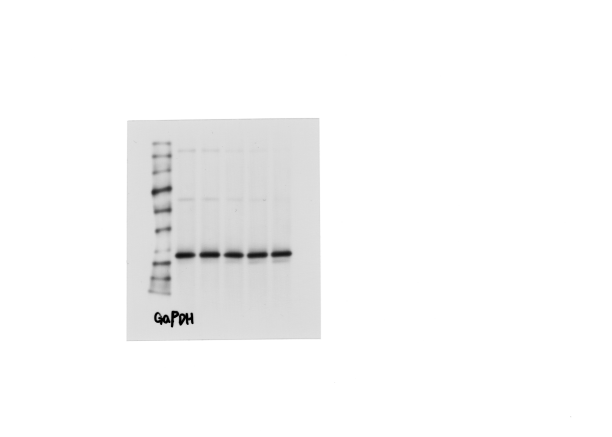 | 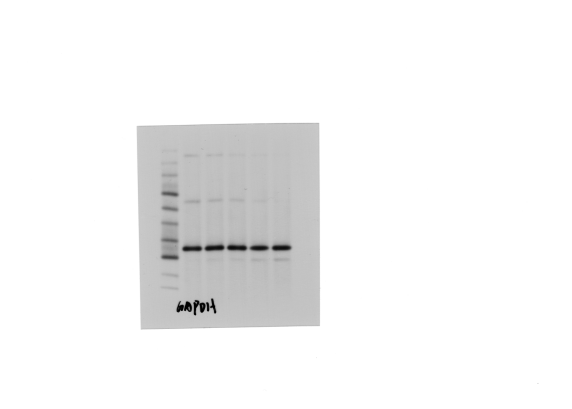 | 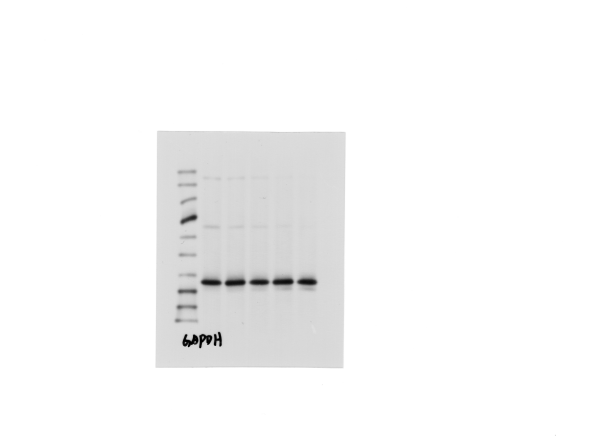 |

Figure S4. The images of the original western blots of proteins.
